# A Dynamic Virtual Channel Approach to Enhance Retinal Prosthetic Precision

**DOI:** 10.3390/biomimetics11050307

**Published:** 2026-05-01

**Authors:** Zhengyang Liu, Tianruo Guo, Yuyan He, Shiwei Zheng, Xiaoyu Song, Cuixia Dai, Jiaxi Li, Xinyu Chai, Yao Chen, Liming Li

**Affiliations:** 1 School of Biomedical Engineering, Shanghai Jiao Tong University, Shanghai 200240, China; liuzy_bme@sjtu.edu.cn (Z.L.); heyy2023@ion.ac.cn (Y.H.); zhengsw@zhejianglab.org (S.Z.); bme_sxy@sjtu.edu.cn (X.S.); seb_li@sjtu.edu.cn (J.L.); xychai@sjtu.edu.cn (X.C.); yao.chen@sjtu.edu.cn (Y.C.); 2 Graduate School of Biomedical Engineering, The University of New South Wales, Sydney, NSW 2052, Australia; 3 College of Sciences, Shanghai Institute of Technology, Shanghai 201418, China; sdadai7412@outlook.com

**Keywords:** artificial vision, epiretinal prosthesis, computational modeling, dynamic virtual channel

## Abstract

Visual prostheses aim to approximate biomimetic visual function by electrically simulating surviving retinal neurons. Improving the spatial resolution of electrically elicited artificial vision remains a critical challenge for retinal prostheses. We investigate how dynamic virtual channel (DVC) parameters shape retinal ganglion cell (RGC) population responses to improve spatial precision and activation efficiency in epiretinal stimulation. We developed a computational modeling framework to quantify DVC performance using a hierarchical optimization strategy. First, static virtual channels (SVCs) were used to map how current ratio (α) and stimulus intensity govern RGC activation, defining an optimal SVC parameter space. Building on this baseline, DVC protocols were refined by evaluating the combined effects of inter-virtual–channel interval (ΔT), α, and intensity. This strategy significantly reduces the complexity of DVC parameter optimization. Under SVC stimulation, increasing intensity improved the linearity of receptive field (RF) centroid displacement with α, while α and intensity jointly set RF centroid location and activated area. Under DVC stimulation, ΔT strongly modulated RGC activation, especially at short intervals. Initializing from SVC-optimized parameters, tuning ΔT and intensity produced more confined activation at lower stimulus intensities than SVC, indicating that DVC can serve as a novel stimulation strategy to enhance spatial precision and activation efficiency in retinal stimulation. This study provides the first systematic analysis of retinal DVC stimulation and a practical optimization framework for next-generation prostheses.

## 1. Introduction

Visual prostheses aim to mimic visual function by recreating (in a simplified form) the retina’s signals. They generate rudimentary visual percepts (phosphenes) by delivering electrical stimulation to surviving inner retinal neurons in patients blinded by degenerative retinal diseases [1,2]. The resulting artificial vision enables implant recipients to perform basic visual tasks such as object localization, motion detection, and simple navigation [3,4,5,6]. Clinical trials of retinal prostheses have demonstrated measurable functional vision, but with clear limitations. Epiretinal devices such as the Argus II (Second Sight Medical Products) enabled ~90% of recipients to perform spatial–motor tasks more accurately than with the system off, with reported grating acuity up to 20/1262 [4,7]. Subretinal systems, including Alpha-IMS/AMS (Retina Implant AG), supported letter reading and motion discrimination, achieving the best Landolt C acuity of 20/546 [8,9]. More recently, the PRIMA photovoltaic implant (Science Corp.) restored form vision with acuity closely matching its 100 μm pixel pitch (~20/420), and, with electronic zoom, improved performance by up to 32 ETDRS letters (~8 lines) over 48 months [10,11]. Suprachoroidal implants offer a less invasive alternative and have shown sustained improvements in object localization, motion discrimination, and daily activities over 2.7 years in patients with advanced retinitis pigmentosa [12].

However, the visual acuity provided by the state-of-the-art retinal prostheses remains constrained to levels within or close to the threshold of legal blindness [10,13,14], reflecting a persistent gap between the fine-grained, retinotopically precise code of natural vision and the blurred, non-selective patterns typically evoked by electrical stimulation. Despite these advances, important challenges remain. Epiretinal stimulation often produces elongated percepts aligned with nerve fiber bundles rather than focal phosphenes, distorting retinotopic mapping [15,16,17]. Enhancing the spatial resolution of electrically elicited artificial vision remains a critical challenge in the field.

Virtual channel (VC) technology, also referred to as current steering or the phantom electrode technique, enables the generation of intermediate stimulation sites by exploiting current interactions between neighboring physical electrodes [18,19,20,21,22,23]. VC has successfully expanded the number of discriminable spectral bands in cochlear implant users. The HiRes 120 strategy [24], implemented in the HiFocus^®^ device with just 16 contact electrodes, was designed to create up to 120 distinct spectral bands through current steering. Clinical studies confirmed that this strategy markedly enhances speech perception and music appreciation in implant recipients [25,26]. To address spatial selectivity, current steering has been explored across neural systems. In visual cortex, dual-electrode stimulation within the same layer can shift activation peaks, although cross-layer steering is less controllable [27,28]. In the retina, three-electrode current steering has improved single-cell selectivity, with response linearity dependent on electrode–cell geometry [29], and optimization frameworks based on field superposition have been proposed for patient-specific calibration [30]. Pioneering retinal VC studies have pursued three primary strategies: (1) optimizing stimulation waveforms [31,32], (2) refining electrode geometries [33], and (3) increasing electrode count and density [20,34,35]. Building on this concept, Matteucci et al. developed a quasi-monopolar stimulation paradigm that maintains comparable stimulus focality and overcomes the high activation threshold limitations inherent to the local current recycling strategy [36]. Spencer et al. improved the spatial addressability of VCs by employing up to six electrodes in a suprachoroidal prosthesis [20], enabling two-dimensional control of activation centroids through modulation of inter-electrode current ratios. Lyu et al. designed a novel epiretinal three-dimensional electrode array that can produced converged and controllable VCs by setting appropriate electrode array parameters and stimulation strategies [33]. Song et al. demonstrated that simultaneous optimization of three coupled parameters, pulse waveform, pulse amplitude, and current ratios across electrode pairs, can achieve more focused VC activation profiles in epiretinal stimulation [32]. In addition, the focality of VC-induced electric fields were improved by replacing distant return electrodes with electrodes adjacent to the stimulation sites [34,35]. Taken together, these studies highlight the substantial potential of VC strategies to improve the spatial resolution of retinal electrical stimulation.

Temporal stimulation strategies have also shown promise. Sequential low-amplitude biphasic pulses can enhance RGC firing while preserving spatial localization, reaching near wild-type responses in degenerated retina [37]. Despite advances in retinal VC development, current studies largely neglect the interactions between sequentially delivered stimuli, which we define as the static virtual channel (SVC) paradigm. In contrast, we define the sequential delivery of multiple continuous VC stimuli as dynamic virtual channel (DVC). The rationale for distinguishing DVC from SVC is grounded in the following observations regarding temporal interaction in retinal stimulation. A series of experimental investigations have shown that the inter-stimulus interval plays a critical role in modulating retinal neuronal responses during sequential electrical stimulation. In particular, short inter-stimulus intervals result in the significant suppression of retinal ganglion cell (RGC) activation to subsequent stimuli [38,39], with the strength of this interaction gradually diminishing as the interval increases. Furthermore, altering the inter-stimulus interval not only impacts phosphene perception during retinal electrical stimulation but also influences visual task performance in prosthetic implant users. In particular, when two consecutive stimuli of equal intensity are applied to the same electrode, the localization accuracy of the phosphene elicited by the second stimulus is dependent on the inter-stimulus interval [40]. During the phase-shifted suprathreshold stimulation of adjacent retinal electrodes, the current amplitude necessary to elicit phosphenes of matched perceived brightness depends on the phase interval between stimuli [41]. In simulated prosthetic vision, structured temporal patterns (e.g., checkerboard or circular sequential stimulation) improve task performance compared to simultaneous or random activation [42,43]. At the neurophysiological level, aperiodic stimulation with variable inter-pulse intervals reduces desensitization and improves response consistency, whereas continuous high-frequency stimulation without recovery periods induces strong adaptation [44,45]. Collectively, these findings underscore the necessity of considering temporal dynamics in VC stimulation—a factor explicitly captured by the DVC paradigm but overlooked in conventional SVC approaches.

The advance of DVC has been explored by both cochlear implants and cortex stimulation. Luo and Garrett demonstrated that dynamically modulating current ratios during sequential VC stimulation enabled implant recipients to perceive pitch contours (flat, rising, and falling) [46]. Concurrently, Beauchamp et al. implemented sequential VC stimulation across a cortical electrode array, facilitating more organized and manageable perceptual patterns in both sighted and blind subjects [47]. To our knowledge, the impact of inter-stimulus intervals on the efficacy of VC stimulation in the retina has not yet been explored.

In this study, we performed an in silico investigation to determine how inter-VC intervals (ΔTs), current ratios, and stimulation intensities shape RGC population activation under DVC stimulation. By incorporating the response properties of RGC populations, we optimized DVC stimulation parameters to improve activation efficacy. We developed a two-stage optimization framework in which SVC-derived parameters (intensity and current ratio) served as the baseline, and the ΔT was introduced as an additional temporal degree of freedom for DVC control. This approach extends the stimulation paradigm from isolated static VC activation, which lacks direct clinical applicability, to sequential dynamic modulation. Unlike prior SVC studies that focused solely on spatial current partitioning, our framework revealed how temporal structuring between VCs interacts with retinal biophysics to regulate RGC activation. In addition, a key novelty of this work lies in demonstrating that ΔT was not merely a timing offset but a new controllable dimension for shaping spatial patterns of neural activation. Our findings show that treating ΔT as an explicit control parameter substantially expands the stimulation control space, enhancing the flexibility, precision, and scalability of VC-based modulation. By quantifying population-level RGC responses, we are the first to establish a mechanistic mapping between DVC parameters and emergent spatial tuning, directly linking stimulation timing to the formation and sharpening of retinal output. This parameter–response map is a necessary step toward the rational design of next-generation stimulation strategies that aim to approximate natural retinal signaling, and it provides a new conceptual and methodological foundation for accelerating the clinical translation of VC technology.

## 2. Materials and Methods

### 2.1. Modeling the Electric Field by Epiretinal Electrical Stimulation

**Figure 1:**
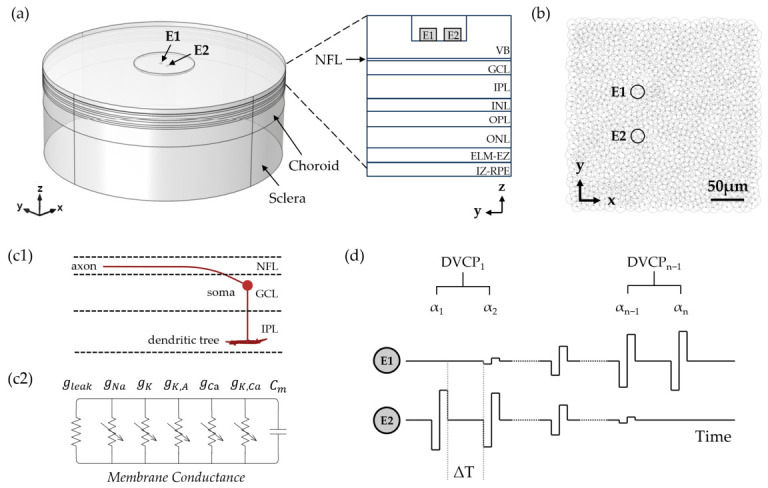
Retinal electrical stimulation model and stimulation paradigm. (**a**) Multilayer finite element model of epiretinal electrical stimulation. A pair of platinum disk electrodes (E1 and E2) were positioned symmetrically about the *x*-axis along the *y*-axis. Their bottom surfaces contacted the vitreous humor, while other surfaces were insulated with polyimide. VB: the vitreous body. NFL: nerve fiber layer. GCL: ganglion cell layer. IPL: inner plexiform layer. INL: inner nuclear layer. OPL: outer plexiform layer. ONL: outer nuclear layer. ELM-EZ: external limiting membrane to the outer border of ellipsoid zone. IZ-RPE: the interdigitation zone to retinal pigment epithelium–Bruch’s complex. (**b**) Spatial distribution of RGC population model in the XY plane, showing soma locations (gray dots) and receptive fields (gray open circles). (**c1**) Morphology and depth distribution of midget RGC model. The RGC model consisted of the soma, axon, and dendritic tree. The axon was divided into four segments ordered by distance from the soma: the initial axon hillock (AH), the high-density sodium channel band (SOCB), the thin section (TS) and the distal axon (DA). (**c2**) Membrane conductance model of the RGC, including the conductance of sodium current ($g_{Na}$), conductance of delayed rectifier potassium current ($g_{K}$), conductance of A-type potassium current ($g_{K,A}$), conductance of calcium current ($g_{Ca}$), conductance of calcium-activated potassium current ($g_{K,Ca}$), and conductance of leak current ($g_{leak}$) and membrane capacitance (C_m_). (**d**) DVC stimulation paradigm. The DVC paradigm consisted of sequential VC stimulation separated by an inter-VC interval (ΔT), defined as the time interval between the offset of the previous stimulus and the onset of the following stimulus. VCs were generated by simultaneously delivering current through two electrodes with specific current ratios. DVCP_1_, … DVCP_n−1_ represent distinct DVC pairs.

The electric field distribution induced by epiretinal electrical stimulation was modeled and calculated using COMSOL Multiphysics 5.6 (COMSOL, Inc., Palo Alto, CA, USA). Electrical stimulation was simulated using the Electric Currents interface in the AC/DC Module of COMSOL Multiphysics^®^. The Dirichlet Condition and Neumann Condition were applied as the boundary conditions [48]. The finite element model of epiretinal electrical stimulation consisted of the vitreous body (VB), the retina, the interdigitation zone to retinal pigment epithelium (RPE)-Bruch’s complex (IZ-RPE), the choroid, the sclera and a pair of stimulating electrodes (E1 and E2) (Figure 1a). The retina was divided into seven layers: the nerve fiber layer (NFL), ganglion cell layer (GCL), inner plexiform layer (IPL), inner nuclear layer (INL), outer plexiform layer (OPL), outer nuclear layer (ONL), and external limiting membrane to the outer border of ellipsoid zone (ELM-EZ). The thickness [49,50] and conductivity parameters [51,52] of each layer were well validated by the literature, with the thickness corresponding to the macular region of the human retina as shown in Table 1. The lower surface of the stimulation electrodes was in direct contact with the vitreous cavity, and the remaining surfaces were insulated with polyimide. The lower surface of the multi-layer model served as the ground and return. The diameter of the stimulation electrodes (Diam) was 20 μm, the distance between the edges of the electrodes (EED) was 50 μm, and the distance from the lower surface of the electrodes to the upper surface of the nerve fiber layer (ERD) was 50 μm. The model used a layer-specific meshing approach, employing an extra-fine mesh in the retina, VB, electrodes, and substrate for accurate potential calculation, and a coarse mesh in the remaining layers (IZ-RPE, choroid, and sclera) to enhance computational efficiency. The lower surfaces of the stimulation electrodes were set as boundary current sources and the stationary study was used to calculate the induced electric field.

### 2.2. Retinal Ganglion Cell Model

The RGC model was established using NEURON simulation environment, version 7.5 (Hines & Carnevale, Yale University, New Haven, CT, USA; http://www.neuron.yale.edu, accessed on 21 April 2026). A population model of 964 RGCs was simulated in a 150 × 150 μm area in a mosaic pattern [53] (Figure 1b). Each RGC consisted of the soma, axon, and dendritic tree. The axon bent at an angle of 45° away from the soma, gradually rising to the nerve fiber layer before becoming horizontal. The axon was further divided into four segments: the initial axon hillock (AH), the high-density sodium channel band (SOCB), the thin section (TS) and the distal axon (DA). The soma size and dendritic field size were modeled based on human midget ganglion cells at a 2 mm eccentricity [54]. The axon, soma, and dendritic field were located within the NFL, GCL, and IPL, respectively (Figure 1(c1)), with the dendritic tree located within 20% to 40% of the IPL thickness [55]. Six types of ion channels were added to the ganglion cell model (Figure 1(c2)): a sodium current ($I_{Na}$), a delayed rectifier potassium current ($I_{K}$), an A-type potassium current ($I_{K,A}$), a calcium current ($I_{Ca}$), a calcium-activated potassium current ($I_{K,Ca}$) and a leak current ($I_{L}$). All six ion channels were distributed across the soma, axon, and dendrite tree, with conductance properties configured according to established literature values [56,57,58]. The maximum conductance of ion channels in different regions of RGC are listed in Table 2. The membrane potential $V_{m}$ of each RGC compartment was governed by the cable equation:

(1) $$\frac{{\partial V}_{m}}{\partial t}=\frac{1}{C_{m}}\left(\frac{1}{R_{a}}\cdot \frac{{\partial^{2}V}_{m}}{\partial x^{2}}+I_{stim}-I_{ion}\right)$$

where $C_{m}$ = 1 μF/cm^2^ is the membrane capacitance, $R_{a}$ = 110 Ω·cm is the axial resistivity, x denotes the spatial coordinate along the longitudinal axis of the compartment (in cm), $I_{stim}$ is the extracellular stimulation current, and $I_{ion}$ is the sum of six ionic currents:

(2) $$I_{ion}=I_{Na}\;+\;I_{K}\;+\;I_{K,A\;}+\;I_{Ca\;}+\;I_{K,Ca\;}+\;I_{L}.$$

Each current was modeled using the Hodgkin–Huxley formalism:

(3) $$I_{Na}={\bar{g}}_{Na}\cdot m^{3}\cdot h\cdot (V_{m}-E_{Na}),$$

(4) $$I_{K\;}={\bar{g}}_{K}\cdot n^{4}\cdot (V_{m}-E_{K}),$$

(5) $$I_{K,A}={\bar{g}}_{K,A}\cdot p^{3}\cdot q\cdot (V_{m}-E_{K}),$$

(6) $$I_{Ca}={\bar{g}}_{Ca}\cdot {c\;}^{3}\cdot (V_{m}-E_{Ca}),$$

(7) $$I_{K,Ca}={\bar{g}}_{K,Ca}\cdot \frac{{\left({\left[Ca^{2+}\right]}_{i}/1\mu M\right)}^{2}}{1+{\left({\left[Ca^{2+}\right]}_{i}/1\mu M\right)}^{2}}\cdot (V_{m}-E_{K}),$$

(8) $$I_{L}={\bar{g}}_{leak}\cdot (V_{m}-E_{L}).$$

The gating variables (m, h, n, p, q, c) follow first-order kinetics:

(9) $$\frac{dx}{dt}=\alpha_{x}(1-x)-\beta_{x}x,$$

where the symbol x generically denotes any of the gating variables m, h, n, p, q, or c, and $\alpha_{x}$ and $\beta_{x}$ are voltage-dependent rate constants. The reversal potentials were $E_{Na}$ = 61.02 mV, $E_{K}$ = −102.3 mV, and $E_{L}$ = −65.02 mV, and the calcium reversal potential $E_{Ca}$ was calculated using the Nernst equation:

(10) $$E_{Ca}=\frac{RT}{2F}\ln\frac{{\left[{Ca}^{2+}\right]}_{o}}{{\left[{Ca}^{2+}\right]}_{i}},$$

where R = 8.314 J·mol^−1^·K^−1^ is the universal gas constant, T = 310.25 K (37.1 °C) is the absolute temperature, F = 96,485 C·mol^−1^ is the Faraday constant, ${\left[{Ca}^{2+}\right]}_{o}$ is the extracellular calcium concentration, and ${\left[{Ca}^{2+}\right]}_{i}$ is the intracellular calcium concentration (in mM). In the NEURON implementation, the Nernst potential is automatically updated as ${\left[{Ca}^{2+}\right]}_{i}$ changes during the simulation. Intracellular calcium dynamics were modeled with a first-order pump:

(11) $$\frac{d{\left[{Ca}^{2+}\right]}_{i}}{dt}=-\frac{3I_{Ca}}{2Fr}-\frac{{\left[{Ca}^{2+}\right]}_{i}-{\left[{Ca}^{2+}\right]}_{rest}}{\tau_{Ca}},$$

where $I_{Ca}$ is the calcium current, F is Faraday’s constant, and r is the depth at which the intracellular calcium concentration is measured. ${\left[{Ca}^{2+}\right]}_{rest}$ represents the resting intracellular calcium concentration, corresponding to the steady-state level maintained in the absence of electrical stimulation, and $\tau_{Ca}$ is the time constant governing the rate of calcium clearance from the intracellular compartment. The temperature of the simulation environment was set to 37.1 °C. More detailed information on the neuron model can be found in our previous work [57].

### 2.3. VC Stimulation Paradigm

In this study, each VC stimulation was generated by simultaneously delivering biphasic charge-balanced pulses to two electrodes, denoted E1 and E2. The current ratio (α) of the VC was defined as the fraction of the total injected current delivered through E1. Accordingly, under VC stimulation, the total current injected into the electrode pair ($I_{total}$) was split between E1 and E2 according to the current ratio α:

(12) $$I_{E1}=\alpha \cdot I_{total},$$

(13) $$I_{E2}=\left(1-\alpha \right)\cdot I_{total}.$$

All electrical stimuli employed in this study were biphasic, cathodic-first, charge-balanced pulses with a pulse width of 0.1 ms per phase. The charge per phase was calculated as $Q=I_{total}\cdot 0.1\;\mathrm{ms}$.

The SVC paradigm generated a single VC independently without considering the time interval between VCs. The DVC paradigm consisted of sequential VC stimulation separated by an inter-VC interval (ΔT), defined as the time between the offset of the previous VC stimulus and the onset of the following stimulus (Figure 1d). To simplify the parameter optimization of DVC stimulation, we proposed a two-step strategy based on optimizing parameters from the SVC stimulation (see Section 3). Under SVC conditions, optimized α values were obtained, and adjacent α values formed dynamic virtual channel pairs (DVCPs).

### 2.4. Evaluation Method for Population Activation Effects

The population response induced by stimulation was quantitatively analyzed using the activated RGC receptive field (RF) area and the centroid of the activated RF region [32,57]. The RF of each RGC was approximated by a circular area, with the size corresponding to the RF size of midget ganglion cells at a 2 mm eccentricity [59,60]. When calculating the activated RF distribution, the contour points of all activated RGC RFs were first extracted, and an elliptical curve fitting was performed based on the contour point data. The area enclosed by the fitted ellipse and the centroid of the ellipse were used as the activated RF and centroid, respectively. The details of the ellipse fitting procedure and the quantitative evaluation of the fitting performance (Figure S5) are provided in the Supplementary Materials. The RGC activation threshold was defined as the minimal total current required to evoke one RGC activation delivered through the electrode pair. A detailed justification for this single-cell threshold definition, along with validation using a population-based criterion, is provided in the Supplementary Materials (Table S5; Figure S3).

## 3. Results

### 3.1. Single-Peak Electric Field Distribution Under Selected Electrode Parameters and Populational Activation Threshold Under SVC

**Figure 2:**
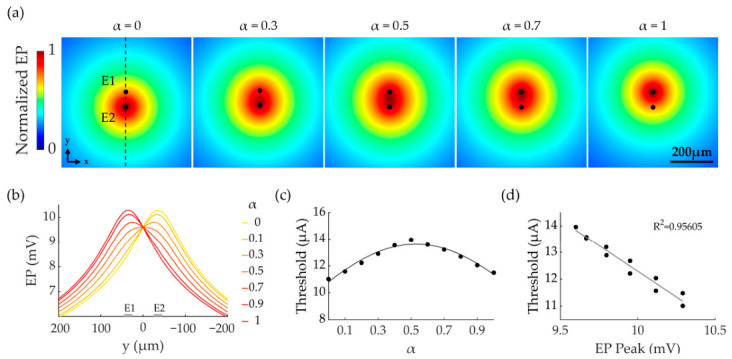
Electric potential (EP) distribution and RGC activation thresholds under SVC stimulation. (**a**) EP distribution on the upper surface of GCL under SVC stimulation at different α values. (**b**) EP profiles along the inter-electrode axis (black dashed line in panel (**a**) as α varied from 0 to 1). For (**a**,**b**), the total current injection on electrode pair was maintained at 1 μA across all α conditions. (**c**) Population activation thresholds in SVC stimulation (STH) as a function of α. Thresholds were defined as the minimal total current injected into the electrode pair required to activate the first RGC. The black curve shows Gaussian fitting of the data points. (**d**) Linear regression between STHs and EP peak values. R^2^ indicates the coefficient of determination for the fit.

As discovered in our previous study, focused SVC stimulation requires a single-peak electric field distribution between the stimulating electrode pair [32,33]. Figure 2a shows the electric potential (EP) distribution generated on the NFL surface by SVC stimulation condition used in this study. The location of the EP peak shifted with α, moving from E2 to the E1 as α increased from zero to one. Figure 2b presents the EP profiles along the inter-electrode axis, demonstrating a unimodal distribution under the selected electrode parameters (20 μm Diam, 50 μm EED, and 50 μm ERD). As α varied from zero to one, the EP peak position progressively shifted from electrode E2 to electrode E1. Under SVC stimulation, the RGC population activation threshold initially rose and then fell with α, peaking at α = 0.5 (Figure 2c). A linear relationship was observed between the EP peak magnitude and the activation threshold, with higher EP peaks corresponding to lower thresholds (Figure 2d).

### 3.2. The Spatial Distribution of Activated RGC RFs Is Shaped by Stimulation Intensity and α Under SVC

**Figure 3:**
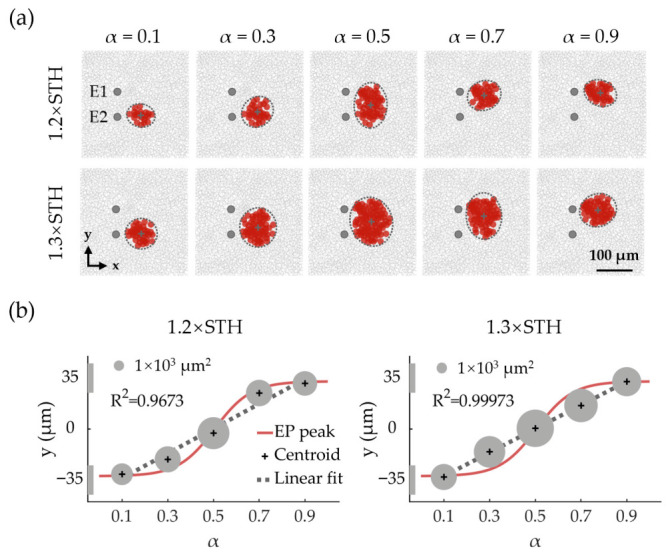
Populational activation of RGCs under SVC stimulation. (**a**) Activation patterns at 1.2× and 1.3× threshold (STH) intensities. Gray crosses mark centroids of activated RGC RFs, with dashed ellipses showing activated RF areas. Red solid circles represented the RFs of the activated RGCs, while inactivated RGCs were indicated in gray open circles. (**b**) α-dependent activated RF profiles at 1.2× and 1.3× STH intensities. Red curve: sigmoidal fit of EP peak locations; black plus signs: activated RF centroids; and gray dashed line: linear fit of RF centroid locations (R^2^, coefficient of determination). Activated RF areas are proportional to circle areas. The two grey rectangles on the y-axis indicate the positions of electrode E1 (positive y-axis) and electrode E2 (negative y-axis).

**Figure 4:**
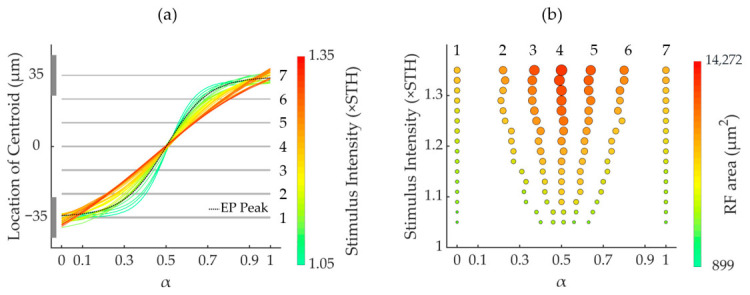
The RGC activation modulated by dual-parameter (α and stimulation intensity) under SVC stimulation. (**a**) Positional changes in EP peaks (black dashed curve) and activated RGC RF centroids (colored curves for different intensities) versus α; all curves were fitted with sigmoidal functions. Gray lines mark sextant divisions between electrodes. The EP was calculated under a total injection current of 1 μA applied to the electrode pair. Although the absolute magnitude of the EP peak varied with current intensity, its peak position remained consistent across different current levels. (**b**) Activated RGC RF areas under stimulus parameter sets (α and stimulation intensity) that positioned their centroids at each sextant line in panel (**a**). Activated RF area is indicated with color. Circle sizes also vary proportionally with RF area for more visually intuitive presentation. STH: population threshold under SVC.

Figure 3 and Figure 4 show the spatial distribution properties of activated RGCs under SVC as the stimulation intensity and α were varied. Stimulation intensity in SVC mode was expressed as multiples of the threshold current (STH). In Figure 3a, under SVC stimulation at identical threshold multiples, the activated RGC RF area initially increased then decreased with α, peaking at α = 0.5. The RF centroid shifted from near electrode E2 toward electrode E1 as α increased. At 1.2× threshold stimulation, centroid movement followed an S-shaped curve relative to α, whereas at the 1.3× threshold, it became more linear (Figure 3b).

Figure 4a shows that across all intensities used in this study, RF centroids consistently migrated from E2 to E1 as α increased. However, the centroid–α relationship varied: lower intensities produced a sigmoidal curve, while higher intensities enhanced linearity. Although higher intensities offered improved centroid control, they also enlarged the activated RGC RF area (Figure 3 and Figure 4), reflecting a trade-off between spatial precision and spread.

To better illustrate the influence of stimulation intensity on activated RF centroids, the space between the E1 and E2 centers was divided by seven equally spaced partition lines. At α = 0.3 (Figure 4a), increasing the intensity from 1.05× to 1.35× STH shifted the centroid from between lines 1 and 2 to between lines 2 and 3. At α = 0.7, the centroid moved from between lines 6 and 7 to between lines 5 and 6 (Figure 4a). These findings revealed that (1) increasing stimulation intensity generally enhances the linearity of centroid displacement as a function of α, and (2) centroid sensitivity to intensity variations is highest near α = 0.3 and 0.7, but minimal at α = 0, 0.5, and 1.

In addition, activated RGC RF areas were also influenced by stimulation intensity under SVC stimulation. Figure 4b presents the integrated results of the stimulus intensity, activated RGC RF area, and α value at a fixed centroid position aligned to partition lines 1–7. The area of activated RGC RFs increased with stimulation intensity, with the rate of increase being more pronounced when α values approached 0.5. To maintain a constant centroid position during stimulus intensity escalation, α values required corresponding adjustments.

To quantitatively characterize the joint effects of α and stimulus intensity, we performed a systematic analysis of the activated RF centroid position and area. As summarized in Table S1 (Supplementary Materials), the overall centroid position shifted monotonically with α across stimulation intensities, with the displacement range varying markedly across α values, e.g., reaching 14.46 μm at α = 0.6, but less than 9 μm near α = 0, 0.5 and 1. The activated RF area increased linearly with stimulus intensity for each α condition (R^2^ > 0.975), with the expansion rate peaking at α = 0.4–0.6 (~35,000 μm^2^/×STH) and dropping to ~18,000–20,000 μm^2^/×STH near α = 0 or 1. Detailed numerical values are provided in Table S1.

### 3.3. Optimized Stimulation Performance Under SVC Stimulation

Under SVC stimulation, activated RF centroids and areas were jointly influenced by stimulation intensity and α in a non-separable manner. Therefore, we proposed an optimization strategy for SVC by optimizing the stimulation intensity and α simultaneously to achieve optimal VC stimulation, defined as producing linear centroid displacement between the stimulating electrodes while maintaining consistent activated RGC RF area sizes.

**Figure 5:**
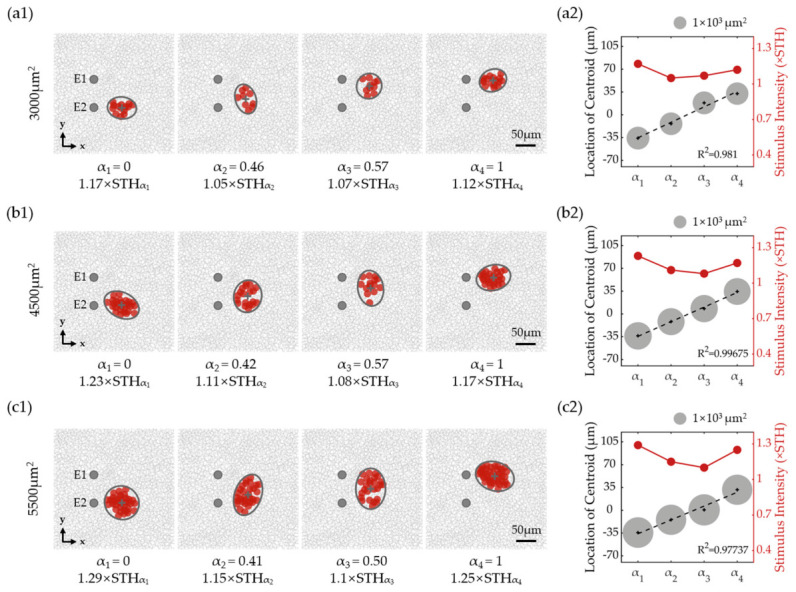
RGC activation results under optimized SVC stimulation parameters. (**a1**) RGC spatial responses elicited by optimized stimulation parameters for target RF area of 3000 μm^2^. The activated RF area was fitted and calculated with ellipse (black line). Gray crosses mark centroids of activated RGC RFs. Red solid circles represented the RFs of the activated RGCs, while inactivated RGCs were indicated in gray open circles. (**a2**) Quantitative characterization of RGC responses shown in (**a1**). Left axis: Activated RGC RF centroid (black crosses) shifts with linear fit (dashed line); RF areas (gray solid circles) were proportional to circle area. Right axis: Applied stimulation intensities for each α value. (**b**,**c**) Results for target RF areas of 4500 μm^2^ and 5500 μm^2^, respectively; the panel layout and descriptions are identical to those in (**a**).

As illustrated in Figure 4a, each partition line (1–7) intersected all centroid–α trajectories across stimulation intensities (from 1.05× to 1.35× STH), indicating that a specific α existed at each intensity in order to position the activated RGC RF centroid on a target line. By selecting appropriate intensity–α pairs at these intersections (Figure 4b), both the RGC RF centroid location and RF area can be simultaneously optimized. Based on this analysis, three optimized stimulus parameter sets were identified, with the results shown in Figure 5a–c for the mean target RGC activation areas of 3000, 4500, and 5500 μm^2^, respectively. The acquisition of these optimal parameters resulted from a trade-off between area consistency and centroid linearity. Across all area groups, the activated RGC RF areas maintained inter-positional differences below 200 μm^2^ while achieving maximal centroid proximity to partition lines 1, 3, 5, and 7 in Figure 4a, confirming a bottom-to-top linear shift with increasing α.

### 3.4. The Baseline Parameters for DVC Stimulation Were Defined Using the Optimized Stimulation Parameters Derived from SVC Stimulation

When the stimulation paradigm was advanced from independent SVC to sequential DVC with VC mutual interaction, the multitude of parameters (α, stimulation intensity, and ΔT) made the RGC regulatory of DVC highly complex. Therefore, we adopted the optimized parameters from SVC conditions as the baseline, and subsequently investigating the effects of ΔT under DVC stimulation. Since smaller activated RGC RF areas correspond to higher stimulation resolution, the 3000 μm^2^ condition in Figure 5 was selected as the optimal SVC outcome. No viable parameter combinations were found that could elicit a smaller RGC activation area while simultaneously satisfying the dual criteria of RF area consistency and linear centroid displacement. We defined the stimulation intensity at each α condition (α_1_ = 0, α_2_ = 0.46, α_3_ = 0.57 and α_4_ = 1) in this group as the base intensity (IB_i_, i = 1, 2, 3, 4; total current injected into the electrode pair) for DVC stimulation: IB_1_ = 1.17 × STH_α1_, IB_2_ = 1.05 × STH_α2_, IB_3_ = 1.07 × STH_α3_, and IB_4_ = 1.12 × STH_α4_ (STH_α1_ = 11.01 μA, STH_α2_ = 13.94 μA, STH_α3_ = 13.73 μA, and STH_α4_ = 11.48 μA). More specifically, the total current injected into the electrode pair (E1 and E2) at base intensity with α_1_, α_2_, α_3_ and α_4_ was 12.88 μA, 14.64 μA, 14.69 μA and 12.86 μA, respectively. By pairing adjacent α values from the optimized SVC stimulation, we established three DVC pairs (DVCPs): α_1_-α_2_ (DVCP_1_), α_2_-α_3_ (DVCP_2_) and α_3_-α_4_ (DVCP_3_). Each DVCP corresponded to a pair of IB_i_ (i = 1,2, 3, or 4). For example, for DVCP_1_ at 1 × IB_i_ stimulation, the stimulation protocol consisted of an initial α_1_ stimulus with intensity IB_1_ (1.17 × STH_α1_) and, after a time interval of ΔT, a subsequent α_2_ stimulus at IB_2_ (1.05 × STH_α2_). When modifying stimulation intensity in DVC, both VCs in a DVCP were scaled equally. We define N as the scaling factor applied equally to the base intensities (IB_i_) of both VCs within a given DVCP. Accordingly, the actual stimulation intensity for each VC was calculated as N × IB_i_, where IB_i_ are the base intensities listed in Table 3 for each α condition. In the present study, N varied from 0.88 to 0.98. The detailed stimulation parameters for each DVCP are specified in Table 3.

### 3.5. Mechanistic Rationale for Using SVC-Optimized Parameters as DVC Initialization

Direct optimization of DVC over the full parameter space (α, intensity, ΔT, and α-pair configuration) is computationally prohibitive due to strong nonlinear coupling among these variables. We therefore adopted a two-stage strategy. First, under SVC conditions, we identified (α, intensity) pairs that produce linear RF centroid displacement with consistent RF areas. These optimized parameters define a low-dimensional subspace that satisfies the primary spatial constraints. Second, within this subspace, we introduced ΔT as an additional temporal degree of freedom and fine-tuned intensity to achieve DVC optimization. This decomposition is mechanistically justified because SVC parameters (α and intensity) primarily govern the spatial characteristics of activation (centroid location and RF area), whereas ΔT primarily modulates temporal integration of membrane potentials without fundamentally altering the spatial activation envelope. As demonstrated in Figure S1 (Supplementary Materials), the RF centroid under DVC remains confined within the spatial envelope defined by the union of the corresponding SVC RFs across a wide range of ΔT and intensity, and the linearity of centroid displacement across DVCPs remains robust (R^2^ > 0.9; Figure S2). Thus, initializing DVC with a SVC-optimized parameters is both computationally efficient and mechanistically grounded.

### 3.6. Stimulation Performance Under DVC Stimulation

**Figure 6:**
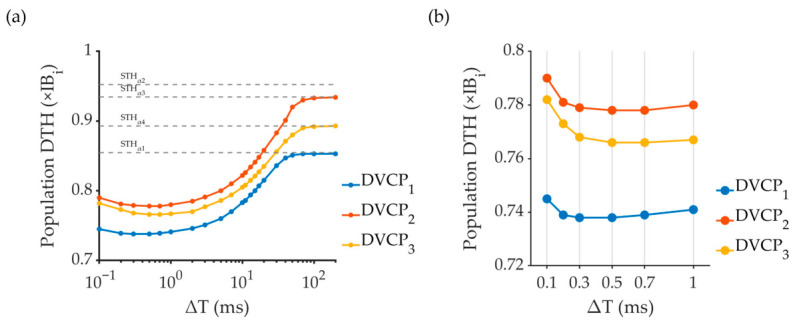
RGC population activation thresholds under DVC stimulation. (**a**) Gray dashed lines indicate population activation thresholds relative to IB_i_ under four SVC stimulation (α_1_–α_4_): STH_α1_ = 0.85 × IB_1_, STH_α2_ = 0.95 × IB_2_, STH_α3_ = 0.93 × IB_3_, and STH_α4_ = 0.89 × IB_4_. (**b**) Enlarged view of population activation thresholds under DVC from 0.1 to 1 ms.

Figure 6 displays DVC thresholds across different ΔTs. The RGC population threshold for DVC stimulation (DTH) at each ΔT was defined as the minimum IB_i_ multiple required to activate the first neuron at the corresponding ΔT when delivered through the electrode pair. As the ΔT increased from 0.1 to 200 ms, the DTH exhibited a triphasic pattern, with an initial slight decline followed by an obvious rise, eventually reaching a stable maximum value. The minimum DTH was observed within the ΔT range of 0.3–0.7 ms. At sufficiently large ΔT values (>100 ms), the DTH curve asymptotically approached and maintained a stable maximum value. The maximum DTH value for each DVCP curve aligned with the lower threshold of its constituent SVCs. At relatively shorter ΔT, DVC stimulation leveraged temporal summation of membrane potentials, enabling RGC activation through paired subthreshold VCs. This integrative effect was consistently observed at the population level as shown below.

**Figure 7:**
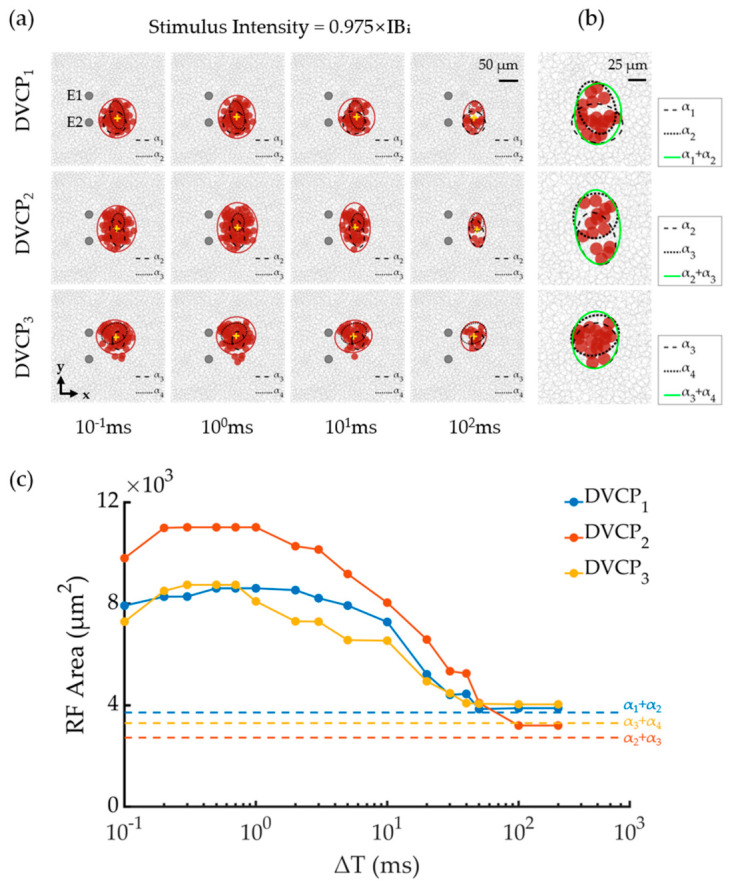
Activated RGC RFs under DVC stimulation with current intensity at 0.975 × IB_i_. (**a**) Activated RFs under DVC stimulation at ΔT = 0.1, 1, 10, and 100 ms. The activated RF areas were calculated from fitted ellipse areas. Elliptical fits for DVCP-activated RFs are shown in red circles, and yellow crosses for the RF centroids. Black dashed ellipses show the fitted RFs elicited by SVC stimulations at the same current intensity (0.975 × IB_i_) for each constituent α. Red solid circles represented the RFs of the activated RGCs, while inactivated RGCs were indicated in gray open circles. (**b**) The calculation method of the union of the RF areas evoked by two SVC stimuli. Sparse and dense dashed ellipses indicate the elliptical fits of the RFs activated by the first and second SVC stimuli in the DVCP, respectively. The green solid ellipse represents the elliptical fit of the combined activation area of the two SVC stimuli. The area of the fitted ellipse was taken to represent the union of RF area activated by the two SVC stimuli. (**c**) ΔT-dependent changes in activated RF areas under DVC stimulation. Colored dashed lines indicate the union of RF areas from corresponding SVC stimulations at 0.975 × IB_i_ (i = 1,2,3 and 4 for α_1_, α_2_, α_3_ and α_4_, respectively).

We then explored the spatial distribution of RGCs activated by DVC stimulation. Figure 7 presents RGC RFs activated by DVC stimulation at a constant current intensity (0.975 × IB_i_). Figure 7a shows representative examples of the activated RFs under DVC stimulation at ΔT = 0.1, 1, 10, and 100 ms. Figure 7c demonstrates the ΔT-dependent modulation of activated RGC RF areas across all DVCP conditions used in this study. Activated RF areas exhibited a triphasic response, increasing with ΔT up to 0.3–2 ms, then decreasing pronouncedly, and eventually reaching a stable value at longer ΔT.

Under small ΔT conditions, adjacent VC stimuli induced strong temporal summation of membrane potential, expanding activated RGC RF areas. When ΔT was below 40 ms, activated RGC RF areas at 0.975 × IB_i_ under DVC stimulation markedly exceeded the combined areas of corresponding SVCs at 0.975 × IB_i_ (dashed lines in Figure 7c). As ΔT further increased, the activated RF area eventually converged to the combined activation area of the two corresponding SVC conditions. The ΔT required for DVCP activation to approach the stable value was approximately 50 ms for DVCP_1_ and DVCP_3_, and 100 ms for DVCP_2_ at 0.975 × IB_i_. Upon examination of all the data, we found that the ΔT required for the RF area evoked by DVCP stimulation to reach a stable value was influenced by both stimulation intensity and α-pair. These findings demonstrated that under DVC stimulation, activated RGC RF areas can be modulated across a wide dynamic range by adjusting ΔT without altering stimulus intensity.

We further quantified the combined effects of ΔT and stimulus intensity on DVC-evoked RF areas. As shown in Table S2 (Supplementary Materials), under a fixed intensity, ΔT exerted a non-monotonic modulation. The activated RF area typically increased from ΔT = 0.1 ms to 1 ms. The magnitude of this initial rise was intensity-dependent: it was most evident at moderate intensities, with, for instance, gains of 19.3% and 11.0% at 0.9 × IB_i_ and 0.95 × IB_i_ (DVCP_1_). In contrast, lower (0.85 × IB_i_) and higher (1 × IB_i_) intensities yielded smaller relative changes (e.g., 6.8% and 5.9% for DVCP_1_, respectively). At small ΔT values, strong temporal summation expanded the RF area to up to 261% of the corresponding SVC union area (DVCP_3_, 1 × IB_i_, ΔT = 1 ms); as ΔT increased from 1 ms to 10–20 ms the area dropped sharply (e.g., DVCP_1_ at 1 × IB_i_: from 10,125 μm^2^ to 7730 μm^2^), and beyond 100 ms, it stabilized near the SVC union area. Reducing stimulus intensity markedly decreased the activated area (e.g., DVCP_1_ at ΔT = 10 ms: from 7929 μm^2^ at 1 × IB_i_ to 1632 μm^2^ at 0.85 × IB_i_), and also amplified the modulatory effect of ΔT (relative area change of 93.7% at 0.85 × IB_i_ vs. 52.1% at 1 × IB_i_ when ΔT increased from 1 to 50 ms). Complete parameter sets are provided in Table S2. It should be noted that for irregular or multi-cluster activation patterns, the ellipse fit provides only a moderate approximation of the underlying spatial distribution; therefore, area values reported under such conditions should be interpreted with caution.

**Figure 8:**
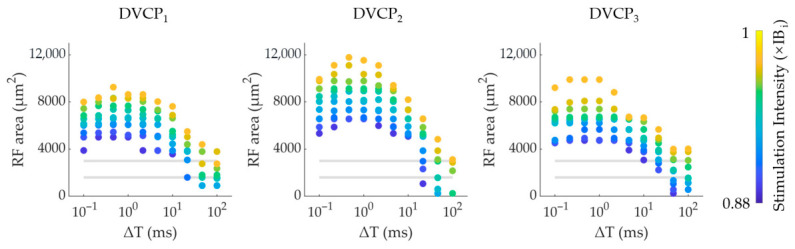
Dual-parameter regulation of activated RGC RF area under DVC stimulation (0.88–0.98 × IB_i_). The gray lines indicate RF area level of 1600 μm^2^ and 3000 μm^2^.

In the DVC stimulation, the activated RGC RF area was jointly modulated by ΔT and stimulus intensity. We computed the activated RGC RF area variation curves for each DVCP across ΔT values (0.1–100 ms) at intensities from 0.88 × IB_i_ to 0.98 × IB_i_ (Figure 8). As shown in Figure 8, lowering intensity shifted the RF area–ΔT curves downward. The modulable range of RGC RF areas varied across DVCP conditions, with DVCP_2_ exhibiting the broadest RF area modulation capacity. Furthermore, the modulative range of RF area also varied with ΔT. It is worth noting that the triphasic ΔT modulation pattern (increase-decrease-plateau) was most pronounced within the intensity range of 0.88–0.98 × IB_i_ (Figure 8). At higher or lower intensities, this pattern became less distinct.

### 3.7. Optimized Stimulation Performance Under DVC Stimulation

**Figure 9:**
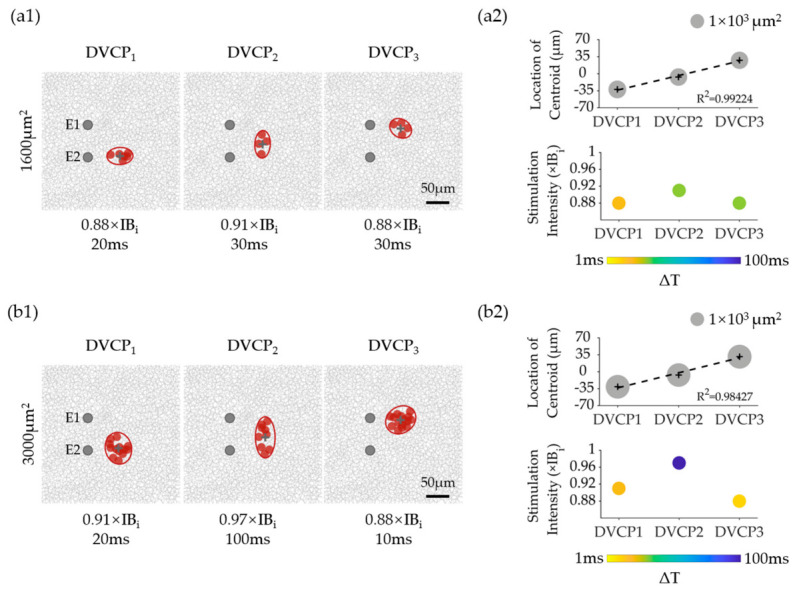
RGC activation results using optimized DVC stimulation parameters. (**a1**) RGC spatial responses elicited by optimized stimulation parameters for target RF area of 1600 μm^2^. Red ellipses indicate the fitted RFs under DVC stimulation, and gray crosses indicate the RF centroids. Red solid circles represented the RFs of the activated RGCs, while inactivated RGCs were indicated in gray open circles. (**a2**) Quantitative characterization of RGC responses shown in (**a1**). Top: Activated RGC RF centroid (black crosses) shifts with linear fit (dashed line) and RF areas (gray solid circles) were proportional to circle area. Bottom: Optimized DVC parameter combinations. (**b**) Results for target RF area of 3000 μm^2^; the panel layout and descriptions are identical to those in (**a**).

Building upon the results in Figure 8, we optimized stimulation parameters (stimulation intensity and ΔT) to achieve satisfying DVCP performance. We selected 1600 and 3000 μm^2^ as the target areas for DVC parameter optimization. The optimized DVCP parameters in Figure 9 exhibited ideal VC performance for both target areas: (1) consistent activated areas (within ±5% of the target area) and (2) linear shifts (R^2^ > 0.98) in the centroid location across different DVCPs. For example, in Figure 9(a1,a2), by choosing appropriate low stimulation intensities (0.88~0.91 × IB_i_) and ΔTs (20~30 ms), the activated RGC RF areas demonstrated a robust size consistency (1584 μm^2^ ± 43 μm^2^) and linear shift in centroid across all DVCPs. A different set of stimulation intensities (0.88–0.97 × IB_i_) and ΔTs (10–100 ms) also produced similar performance for the 3000 μm^2^ target area. The RGC RF areas elicited under optimized DVC parameters were smaller than those elicited by the best-performing SVC stimulation, highlighting the advantages of DVC stimulation in achieving higher-resolution electrical stimulation. Notably, to achieve the target RF areas (e.g., 1600 μm^2^ or 3000 μm^2^), the optimized DVC parameters consistently required lower stimulation intensities compared to the best-performing SVC stimulation. This indicates that DVC offers improved stimulation efficiency—producing comparable or more confined activation with less injected charge. It is important to note that when stimulus intensity was matched to the optimal SVC level (1 × IB_i_), DVC did not reduce the activated RF area relative to SVC; at short ΔT, the area was in fact considerably larger (Figure S4). The smaller RF areas achieved by DVC in Figure 9 therefore reflect the combined benefit of operating at lower intensities together with tuned ΔT. Additionally, the spatial selectivity of DVC stimulation was evaluated using the full RGC coordinate data. The mean activation threshold of RGCs within the target region was found to be 0.86–0.94 times that of RGCs in the surrounding annular region. The detailed methodology and results are provided in the Supplementary Materials (Figure S6).

## 4. Discussion

In this study, we explored the potential of DVC stimulation to advance retinal neuro-prosthetic performance. While previous work has validated the feasibility of SVCs, our study extended the virtual–channel paradigm from independent, static stimulation to the sequential delivery of temporally structured VCs. By systematically examining how stimulation parameters regulate RGC responses under both SVC and DVC conditions, we identified optimal strategies for improving population-level activation. Two key findings emerged: (1) the inter-VC interval (ΔT) exerted a strong and previously uncharacterized influence on RGC population activation, and (2) appropriately configured DVC parameters can produce more spatially focused population responses at lower activation thresholds than SVC stimulation. Together, these results demonstrated the functional advantages of DVC stimulation and established a more comprehensive theoretical framework to guide the future clinical translation of VC-based retinal prosthetic strategies.

### 4.1. DVCs Can Serve as a Novel Stimulation Strategy for Retinal Stimulation

DVC stimulation has been shown in previous studies to enhance functional restoration across multiple prosthetic applications. For example, Beauchamp et al. demonstrated successful shape perception elicitation in the primary visual cortex using DVC stimulation, despite patient-specific variations in stimulation intervals (ranging from 8.3 to 2000 ms) and sequence lengths [47]. These findings highlighted the importance of selecting appropriate inter-stimulus intervals to facilitate shape perception. In epiretinal prostheses, sequential stimulation across 3–4 electrodes yielded significantly better shape recognition compared to synchronous stimulation [61]. Similarly, in cochlear implants, VC approaches expanded the pitch perception range, while DVC strategies enabled pitch contour identification within extended frequency bands [46]. Consistent with these findings, our computational study indicated that DVC stimulation can similarly improve retinal activation performance, supporting its potential as a novel and more effective stimulation strategy for retinal prostheses.

Our work is the first systematic analysis of temporal modulation at the RGC population level. Previous studies on temporal stimulation primarily evaluated behavioral or perceptual outcomes (e.g., improved discrimination or recognition under sequential stimulation [42,43,61]). While these works demonstrate functional benefits, they do not establish a quantitative relationship between temporal parameters and underlying neural responses. In contrast, our study provides the first systematic, model-based analysis of how inter-stimulus timing (ΔT) modulates RGC population activation, thereby offering a mechanistic foundation linking temporal stimulation strategies to observed perceptual improvements.

DVC extended the VC stimulation into the temporal domain. Prior VC studies have focused on optimizing spatial and electrical parameters, including waveform design, electrode geometry, and current ratios [20,32,33,36,62,63]. These approaches treat VCs as static entities and do not account for temporal interactions between sequential stimuli. In contrast, we extend the VC framework to DVC by introducingΔT as an additional control dimension. This enables the sequential modulation of VCs, providing greater flexibility in shaping neural activation. We further propose a systematic optimization framework for DVC, demonstrating improved stimulation efficiency and controllability compared to conventional SVC.

Achieving reliable form perception in visual prostheses requires phosphenes with consistent sizes across stimulation sites [61]. In addition, maintaining the linear spatial displacement of RF centroids between adjacent DVCPs is a critical design requirement for VC strategies aimed at improving spatial selectivity. Accordingly, the objective of DVC optimization is to generate multiple RGC activation regions that exhibit (1) comparable RF areas and (2) near-linear centroid displacement across DVCPs. Building on the optimized SVC parameters, we further jointly optimized ΔT and stimulation intensity within the DVC paradigm to identify stimulation conditions that reduced RGC RF areas while preserving linear centroid shifts across DVCPs. This combination of tighter spatial confinement and predictable positional mapping may support more stable phosphene shapes and improved functional outcomes in visual prosthetic systems, thereby advancing the precision and functional fidelity of artificial vision systems.

### 4.2. The Inter-Stimulus Interval Significantly Influences Population Activation Patterns of RGCs Under DVC

Under DVC stimulation, RGC population activation thresholds exhibited a clear dependence on ΔT, with an initial decrease followed by a monotonic increase that stabilized at longer ΔTs, ultimately approaching the lower SVC threshold among the constituent α conditions (Figure 6). This threshold modulation mirrors findings in cochlear implant studies, where perceptual thresholds were minimized at short inter-pulse intervals (0.2–1.1 ms) due to membrane potential temporal summation [23,64]. Consistent with this mechanism, temporal summation in our model peaked at ΔT values of 0.3–0.7 ms, weakened with increasing ΔT, and ultimately dissipated under the present electrode configuration (Figure 6).

This summation effect also exerted strong influence on RGC population activation. At shorter ΔTs, temporal summation recruited additional RGCs, producing larger population responses. Under fixed stimulation intensity, the RF area–ΔT relationship displayed a characteristic triphasic profile—an initial slight expansion, followed by a monotonic reduction, and eventual stabilization at longer ΔTs (Figure 7b,c). Unlike previous epiretinal work reporting no ΔT-dependent differences in shape recognition [61], our results showed clear ΔT-dependent RF modulation, likely due to our close electrode spacing (50 μm vs. 200 μm clinically), which enabled stronger neural summation.

### 4.3. DVC Achieves More Flexible Modulation of RF Activation Areas with Lower Thresholds than SVC Stimulation

DVC stimulation offers clear advantages over SVC in both efficiency and spatial precision. First, DVC required lower intensities to activate RF areas comparable to SVC (Figure 5a and Figure 9b) because sequential pulses produced temporal summation, allowing subthreshold depolarizations to accumulate and reach the activation threshold without high-amplitude pulses. Second, optimized DVC parameters yielded markedly smaller RF areas (1584 ± 43 μm^2^) than the best SVC configuration (3036 ± 57 μm^2^; Figure 9a). Critically, this improvement does not arise from ΔT modulation alone at a matched current. As shown in Figure S4, when current is held constant at the SVC optimum (1 × IB_i_), DVC activation areas are greater than or comparable to those of SVC, depending on the value of ΔT. The enhanced spatial confinement was achieved by jointly lowering stimulus intensity and optimizing ΔT. This outcome reflects the principal advantage of DVC over conventional SVC stimulation: an expanded parameter control space enabled by the simultaneous tuning of intensity and ΔT. It should be noted that while DVC stimulation exhibits genuine spatial selectivity (Figure S6), the mean activation threshold within the target region is approximately 0.86–0.94 times that of the surrounding annular region, the effect is modest in absolute magnitude. Taken together, these features demonstrate that DVC can achieve efficient, focused activation while reducing overall charge delivery—an advantage for energy-efficient, high-resolution retinal prostheses.

### 4.4. This Study Proposes a Novel Methodology for Investigating DVC

Although this study applied DVC stimulation at only three sites (Figure 9), it established a framework for broader exploration. Increasing the number of α-pair combinations could enable finer spatial targeting. Scaling DVC to array-based stimulation will require consideration of (i) spatial constraints among electrode size, spacing, and electrode–retina distance that determine VC convergence, and (ii) electrode-pair alignment relative to RGC axon trajectories. Preliminary constraints for VC convergence have been reported in our earlier work [32,33,65]. With respect to point (ii), a previous study has demonstrated that the relative spatial relationship between electrode pairs and RGC axonal pathways significantly influenced RGC activation patterns [66]. This underscores the need for systematic investigation of electrode-pair alignment in DVC paradigms to better predict and optimize DVC-evoked responses for clinical translation.

### 4.5. DVC Serves as a More Clinically Translatable Stimulation Strategy Beyond SVC

The DVC approach offers greater clinical translatability than SVC. In this study, we advanced the stimulation paradigm from independent, static VC activation to sequential VC modulation, thereby creating DVC that more closely approximates clinical conditions. In our previous study, the temporal interval and mutual interaction between successive VC stimulations were not considered [32]. As a result, this stimulation protocol was not directly applicable to clinical practice. However, it remains a useful mechanistic reference and provides baseline parameters for optimizing DVC strategies.

Although transitioning from SVC to DVC stimulation introduced only one new parameter, the temporal interval (ΔT) between VCs, our model revealed that the modulation of RGC responses under DVC conditions became substantially more complex. Variations in any single DVC parameter, such as the alpha value, ΔT, alpha pair configuration, or stimulus intensity, can alter both the RF area and the centroid position of activated RGCs. To satisfy the dual requirement of achieving an approximately linear displacement of RF centroids while preserving near-constant RF areas, an exhaustive combinatorial search across all parameter sets (alpha value, alpha pair, ΔT, and stimulus intensity) was required, rendering DVC optimization highly intricate. To address this challenge, we developed a two-step optimization protocol: (i) Optimizing SVC parameters by adjusting the alpha value and stimulus intensity to achieve a near-linear displacement of RF centroids; and (ii) fine-tuning only ΔT and stimulus intensity under DVC conditions, using the optimized SVC parameters as a foundation, to maintain consistent RF areas. The results demonstrated that the activated RF centroids under DVC stimulation still maintained a well-preserved linear displacement. The establishment of this DVC stimulation optimization strategy substantially reduced the computational workload.

This developed methodology provides a systematic and efficient framework for optimizing DVC stimulation and lays a solid foundation for its future clinical translation. Importantly, a key advantage of DVC is that it is software-defined and does not require hardware modification. Temporal parameters such as stimulus timing are already programmable in existing systems (e.g., Argus II), and recent hardware developments support high-speed multi-electrode control with fine temporal resolution. Therefore, DVC can be implemented as a firmware-level upgrade, providing a practical pathway to improve stimulation efficiency and spatial resolution without redesigning the implant.

### 4.6. Biological and Clinical Relevance of DVC

RGC responses are highly sensitive to temporal stimulation patterns. Prior studies show that continuous or periodic stimulation induces adaptation and reduced firing, whereas introducing temporal gaps or irregular timing preserves responsiveness and improves consistency [45,67]. Additionally, burst or sequential stimulation can enhance firing while maintaining spatial selectivity [37]. These findings support the biological plausibility of DVC, whereΔT modulates neural activation through temporal integration and recovery mechanisms.

Clinical and experimental evidence demonstrates strong spatiotemporal interactions in retinal stimulation. Sequential stimulation at short intervals can alter perceptual thresholds and induce facilitation or suppression effects depending on temporal characteristics [68,69]. Moreover, current prostheses often suffer from poor spatial resolution due to axonal activation and phosphene overlap. Sequential stimulation has been shown to improve perceptual performance by reducing such interference [61]. In this context, DVC provides a principled framework to control both activation strength and spatial extent via ΔT, addressing the key limitations of existing devices. Importantly, DVC also offers flexibility to accommodate patient-specific variability in electrode–retina interface properties, enabling more consistent activation across locations.

### 4.7. SWOT Analysis of DVC

DVC offers several key strengths: it is grounded in RGC temporal integration, enables spatiotemporal modulation via the joint optimization of the current ratio (α) and inter-VC interval (ΔT), and is fully compatible with existing prosthetic hardware, allowing software-level implementation without redesign. It also provides flexibility for patient-specific calibration, helping mitigate electrode–retina variability.

However, DVC introduces increased optimization complexity and currently lacks experimental and clinical validation. Personalized parameter tuning is required due to inter-subject variability.

Opportunities include integration with AI-driven optimization and advanced image processing for improved stimulation strategies. Threats arise from competing technologies (e.g., gene therapy and optogenetics) and the limited commercial viability historically observed in retinal prostheses.

### 4.8. Limitations and Future Work

This study is based on computational modeling and has not yet been validated experimentally. The model includes only a single RGC subtype at a fixed retinal location, and the explored parameter space is limited to selected VC combinations. In addition, outcome metrics (activation threshold and RF area) are indirect proxies, and their relationship to perceptual outcomes remains to be established. Future work will expand to more comprehensive retinal models and pursue ex vivo and in vivo validation.

## 5. Conclusions

This work establishes a conceptual framework for optimizing DVC stimulation as a pathway toward high-resolution retinal prosthetic strategies. Unlike SVC stimulation, where activation patterns are largely governed by stimulation intensity and current ratios, DVC performance is also highly sensitive to temporal dynamics besides stimulation intensity and current ratios. In particular, the inter-stimulus interval between consecutive VCs significantly shapes the spatial extent and focality of RGC activation, especially at shorter intervals where temporal overlap of electric fields becomes pronounced. By systematically refining ΔT and stimulation amplitude, DVC paradigms can be engineered to evoke more spatially selective, energy-efficient population responses that better approximate physiological recruitment, thereby advancing the precision and functional fidelity of artificial vision systems.

## Figures and Tables

**Table 1:** Thickness and conductivity of each layer in the multi-layered epiretinal stimulation model [49,50,51,52].

Layers		Thickness (μm)	Conductivities (S·m^−1^)
VB		-	1.2821
Retina	NFL	5.4	0.0126
	GCL	31.3	0.0126
	IPL	53.1	0.0571
	INL	28.7	0.0147
	OPL	36.3	0.0571
	ONL	49.2	0.0147
	ELM-EZ	32	1.0309
IZ-RPE		31.1	0.0010
Choroid		300	0.2779
Sclera		933	0.5028

**Table 2:** Maximum conductance of ion channels in RGC [56,57,58].

	AH	SOCB	TS	DA	Soma	Dendrite
${\bar{g}}_{Na}$ (10^−3^ S/cm^2^)	70	1050	100	70	80	25
${\bar{g}}_{K}$ (10^−3^ S/cm^2^)	18	70	18	18	18	12
${\bar{g}}_{K,A}$ (10^−3^ S/cm^2^)		54	54		54	36
${\bar{g}}_{Ca}$ (10^−3^ S/cm^2^)		1.5			1.5	2
${\bar{g}}_{K,Ca}$ (10^−3^ S/cm^2^)	0.065	0.065	0.065	0.065	0.065	0.001
${\bar{g}}_{leak}$ (10^−3^ S/cm^2^)	0.005	0.005	0.005	0.005	0.005	0.005

AH—initial axon hillock, SOCB—high-density sodium channel band, TS—thin section, DA—distal axon. ${\bar{g}}_{Na}$—maximum conductance of sodium current, ${\bar{g}}_{K}$—maximum conductance of delayed rectifier potassium current, ${\bar{g}}_{K,A}$—maximum conductance of A-type potassium current, ${\bar{g}}_{Ca}$—maximum conductance of calcium current, ${\bar{g}}_{K,Ca}$—maximum conductance of calcium-activated potassium current, and ${\bar{g}}_{leak}$—maximum conductance of leak current.

**Table 3:** DVC stimulation conditions.

DVC Stim.	DVCP_1_		DVCP_2_		DVCP_3_	
First and Second VC	α_1_	α_2_	α_2_	α_3_	α_3_	α_4_
Stimulation Intensity						
1 × IB_i_	1.17 × STH**_α__1_**	1.05 × STH**_α__2_**	1.05 × STH**_α__2_**	1.07 × STH**_α__3_**	1.07 × STH**_α__3_**	1.12 × STH**_α__4_**
N × IB_i_	1.17 N × STH**_α__1_**	1.05 N × STH**_α__2_**	1.05 N × STH**_α__2_**	1.07 N × STH**_α__3_**	1.07 N × STH**_α__3_**	1.12 N × STH**_α__4_**

N: scaling factor applied to base intensities (IB_i_) for both VCs in a DVCP.

## Data Availability

The original contributions presented in this study are included in the article. Further inquiries can be directed to the corresponding author.

## Supplementary Materials

The following supporting information can be downloaded at: https://www.mdpi.com/article/10.3390/biomimetics11050307/s1. Figure S1: DVCP_3_ activated RGC RFs across different current intensity and ΔT; Figure S2: Evaluation of RF centroid displacement linearity under DVC stimulation; Figure S3: SVC and DVC population thresholds under multi-cell criterion; Figure S4: Activated RGC RFs under DVC stimulation with current intensity at 1 × IB_i_; Figure S5: RF ellipse fitting performance; Figure S6: Activation selectivity measurement under DVC conditions; Table S1: RF centroid position, activated area at 1.1 × STH, and linear fitting parameters (K and R^2^) of area versus intensity under SVC stimulation with varying current ratio α; Table S2: Activated RF area of RGCs under DVC stimulation at different inter-virtual-channel intervals (ΔT) and stimulus intensities, expressed as absolute values and as percentages of the optimal SVC union area; Table S3: Literature directly relevant to retinal prostheses (2023 onwards); Table S4: Literature directly relevant to current steering (2021 onwards); Table S5: Summary of publications utilizing single-cell threshold definition; Table S6: Optimal parameters under SVC and DVC; Table S7: Literature directly relevant to DVC stimulation, temporal interactions in retinal prostheses, and computational RGC modelling. References [70,71,72,73,74,75,76,77,78,79,80,81] are cited in Supplementary Materials.

## Abbreviations

The following abbreviations are used in this manuscript:

DVC	Dynamic Virtual Channel
SVC	Static Virtual Channel
STH	Population Activation Threshold under SVC
DTH	Population Activation Threshold under DVC
IB_i_	Base Intensity
ΔT	Inter-Virtual–Channel Interval
DVCP	Dynamic Virtual Channel Pair
RGC	Retinal Ganglion Cell
RF	Receptive Field
EP	Electric Potential
VB	Vitreous Body
RPE	Retinal Pigment Epithelium
IZ-RPE	Interdigitation Zone to Retinal Pigment Epithelium–Bruch’s Complex
NFL	Nerve Fiber Layer
GCL	Ganglion Cell Layer
IPL	Inner Plexiform Layer
INL	Inner Nuclear Layer
OPL	Outer Plexiform Layer
ONL	Outer Nuclear Layer
ELM-EZ	External Limiting Membrane to the Outer Border of Ellipsoid Zone
Diam	Diameter of the Stimulation Electrodes
EED	Edge-to-Edge Distance between Electrodes
ERD	Electrode–Retina Distance
AH	Initial Axon Hillock
SOCB	High-Density Sodium Channel Band
TS	Thin Section
DA	Distal Axon
$I_{Na}$	Sodium Current
$I_{K\;}$	Delayed Rectifier Potassium Current
$I_{K,A}$	A-type Potassium Current
$I_{Ca}$	Calcium Current
$I_{K,Ca}$	Calcium-Activated Potassium Current
$I_{L}$	Leak Current
$g_{Na}$	Conductance of Sodium Current
$g_{K}$	Conductance of Delayed Rectifier Potassium Current
$g_{K,A}$	Conductance of A-type Potassium Current
$g_{Ca}$	Conductance of Calcium Current
$g_{K,Ca}$	Conductance of Calcium-Activated Potassium Current
$g_{leak}$	Conductance of Leak Current
