# Supplementary material for: A Dynamic Virtual Channel Approach to Enhance Retinal Prosthetic Precision
Source: Biomimetics (Basel). 2026 May 1;11(5):307. doi: 10.3390/biomimetics11050307 (PMC13204981; doi:10.3390/biomimetics11050307)
Supplement: Supplementary file 1 [file biomimetics-11-00307-s001.zip › biomimetics-4218236-supplementary.pdf]

Mechanistic Rationale for using SVC-optimized Parameters as DVC Initialization

i. Computational feasibility and optimization strategy

Direct parametric optimization of DVC stimulation is highly complex. Although DVC introduces only one additional parameter—the temporal interval (ΔT) between VCs—the resulting RGC response becomes substantially more nonlinear and coupled. Specifically, variations in α (current ratio), ΔT, α-pair configuration, and stimulus intensity jointly influence both the receptive field (RF) area and the centroid position of activated RGC populations. Achieving the dual objective of i. the approximately linear displacement of RF centroids and ii. the preservation of near-constant RF areas would therefore require an exhaustive combinatorial search over a high-dimensional parameter space.

To address this, we first identify, under SVC conditions, the combinations of α and stimulus intensity that produce linear RF centroid displacement with comparable RF areas. These SVC-optimized parameters define a low-dimensional subspace that already satisfies the primary spatial constraints. DVC optimization is then performed by introducing ΔT and fine-tuning the stimulus intensity within this constrained space. This sequential strategy effectively decomposes a high-dimensional global optimization problem into two tractable sub-problems, thereby significantly reducing computational cost while preserving solution quality.

ii. Mechanistic rationale based on RGC activation dynamics

The rationale for this initialization is further supported by the distinct roles of SVC and DVC parameters in shaping RGC activation.

Under SVC conditions, α and stimulus intensity primarily determine the *spatial characteristics* of activation, including RF centroid location and RF area (Figures 3–4). Optimizing these parameters ensures the controlled and approximately linear spatial translation of RF centroids across stimulation sites, while maintaining comparable activation strength.

In contrast, DVC introduces ΔT, which primarily modulates the *temporal integration* of membrane potentials. Specifically, ΔT governs the degree of temporal summation across sequential VC stimuli, thereby altering the activation efficiency without fundamentally redefining the spatial activation pattern. As shown in Figure S1, decreasing ΔT enhances temporal summation and increases RF area, whereas increasing ΔT leads to stabilization of activation. Importantly, the RF centroid remains confined within the spatial envelope defined by the union of the corresponding SVC RFs, with only minor shifts observed.

Furthermore, the linearity of RF centroid displacement across DVC pairs remains robust under variations in ΔT and stimulus intensity, with the coefficient of determination (R^2) consistently exceeding 0.9 (Figure S2). This indicates that temporal modulation via ΔT preserves the spatial mapping established under SVC optimization. Therefore, SVC optimization establishes the spatial framework of RGC activation, while DVC parameters provide temporal modulation within this framework. Using SVC-optimized parameters as initialization is thus both mechanistically justified and computationally efficient, enabling the reliable and tractable optimization of DVC stimulation.

Quantitative characterization of how current ratio (α) and stimulus intensity regulate the activated RF centroid position and area under SVC conditions.

i. Regulation of centroid longitudinal position. As α increased from zero to one, the longitudinal coordinate of the centroid (Y-direction) shifted progressively from negative values (near electrode E2) to positive values (near electrode E1), indicating that α directly governs the longitudinal displacement of the activation region. The range of centroid longitudinal displacement (Range of Centroid Y) under each α condition varied markedly across different stimulation intensities. Within the ranges of α = 0.2-0.4 and α = 0.6-0.7, the centroid exhibited a relatively large longitudinal shift (e.g., reaching 14.46 μm at α = 0.6), indicating that variations in stimulus intensity can induce more pronounced centroid displacements under these α values. In contrast, near α = 0, 0.5 and 1.0, the centroid displacement range was considerably smaller (approximately 5-9 μm), indicating that the centroid position is less sensitive to stimulus intensity at these α values.

ii. Regulation of activated RF area. At a stimulus intensity of 1.1× STH, the activated RF area initially increased and then decreased as α increased, reaching a peak value of 5576.78 μm^2 at α = 0.5. This pattern is consistent with maximal electric field superposition at the midpoint between the two electrodes, where the injected currents are equal and the spatial spread of the electric field is greatest. Across all α conditions, the RF area exhibited a monotonic increase with stimulus intensity. We performed linear regression on the RF area versus stimulus intensity relationship for each α condition, according to the following equation:

$$y = K \cdot (x - 1.1) + A$$

where x denotes the stimulus intensity (×STH), y denotes the activated RF area (μm^2), the parameter K represents the average increment in RF area per unit increase in stimulus intensity (μm^2/×STH), and A corresponds to the activated area at the minimal tested intensity (1.1×STH). All fits yielded coefficients of determination R^2>0.975, confirming a robust linear relationship within the examined intensity range. The rate at which the activated area expanded with increasing stimulus intensity was quantified by the slope K. The K values exhibited a similar distribution pattern, peaking in the middle and tapering toward both ends. Specifically, K reached its maximum (approximately 35,000 μm^2/×STH) within the range of α = 0.4-0.6, indicating that the activated area is most sensitive to changes in stimulus intensity in this region, where even a modest increase in intensity leads to a pronounced expansion of the activated region. In contrast, K values were considerably smaller near α = 0 or α = 1 (approximately 18,000–20,000 μm^2/×STH), suggesting that when stimulation is

delivered closer to physical electrodes, the spatial spread of activation is more constrained and thus more controllable.

Effects of stimulus intensity and inter-virtual-channel interval (ΔT) on activated RF area under DVC stimulation (Table S2).

i. Modulation of activated RF area by inter-virtual-channel interval ΔT. Under fixed stimulus intensity, ΔT exerted a non-monotonic modulatory effect on the DVC-activated RF area (Figure 7). The activated RF area typically increased from ΔT = 0.1 ms to 1 ms. The magnitude of this initial rise was intensity-dependent: it was most evident at moderate intensities with, for instance, gains of 19.3% and 11.0% at 0.9×IB_i and 0.95×IB_i ($DVCP_1$). In contrast, lower (0.85×IB_i) and higher (1×IB_i) intensities yielded smaller relative changes (e.g., 6.8% and 5.9% for $DVCP_1$, respectively). At ΔT = 1 ms, both the absolute activated area and its percentage relative to the SVC union area remained at elevated levels (e.g., reaching 230% for $DVCP_1$ at 1×IB_i), indicating that the two pulses delivered at extremely short intervals induced a strong temporal summation effect, thereby recruiting additional RGCs. As ΔT increased from 1 ms to 10–20 ms, the activated area declined sharply. For instance, under 1×IB_i stimulation, the activated RF area of $DVCP_1$ decreased from 10,125 μm^2 to 7929 μm^2, with the corresponding percentage dropping from 230% to 180%. When ΔT exceeded 100 ms, the activated area stabilized and approached the SVC union area (with the percentage approaching or reaching 100%), indicating that the temporal interaction between pulses had largely dissipated.

ii. Modulation of activated RF area by stimulus intensity. Under a fixed ΔT, reducing the stimulus intensity led to a marked decrease in the activated area. For example, in $DVCP_1$ at ΔT = 10 ms, lowering the intensity from 1×IB_i to 0.85×IB_i reduced the activated area from 7929 μm^2 to 1632 μm^2, corresponding to a decrease of approximately 79%. At lower intensities (0.85–0.90×IB_i), several long-ΔT conditions failed to activate any neurons (empty cells in the table). Furthermore, the modulatory effect of ΔT was more pronounced at lower intensities. Taking $DVCP_1$ as an example, when ΔT was extended from 1 ms to 50 ms at 0.85×IB_i, the activated area shrank from 3878 μm^2 to 246 μm^2, representing a relative change of 93.7%. In contrast, at the higher intensity of 1×IB_i, the corresponding area reduction was only 52.1% (from 10,125 μm^2 to 4845 μm^2).

Rationale for Defining Threshold Based on a Single-Cell Criterion

In the present work, we adopted the single-cell threshold for two main reasons. First, it is a widely established definition in both computational and experimental RGC studies, where threshold is defined as the minimum current required to elicit an action potential in a neuron, typically determined with high precision (e.g., Jeng et al., 2011, Tsai et al., 2012, Kish et al., 2023, and Song et al., 2024; see also Table S5 for experimental studies). Second, it provides a consistent and intuitive baseline for comparing stimulation intensity and receptive field (RF) activation across conditions: the 1× threshold corresponds to a comparable minimal activation level. In contrast, defining the threshold based on a fixed number of activated cells (e.g., 10 RGCs) would result in different RF areas across stimulation sites due to spatial variability in RGC distribution, complicating cross-condition comparisons.

To verify the robustness of the main findings, we recalculated the activation thresholds using a population-based definition (minimum current to activate ten RGCs). As shown in Figure S3, the main findings remain unchanged: under SVC, the threshold–α relationship retains a Gaussian profile (Figure S3a), and under DVC, the threshold–ΔT relationship remains triphasic (Figure S3b). These results demonstrate that our conclusions are robust to the choice of threshold definition.

Ellipse Fitting Procedure and Quantitative Assessment of Fitting Performance

To improve fitting robustness, particularly for irregular or multi-cluster activation patterns, the fitting procedure utilizes a three-step selection strategy: i. area-based decision to determine whether multiple regions should be treated jointly; ii. shape–consistency check based on center distance and axis ratio differences; and iii. final selection of the ellipse (global vs. largest-region fit) based on maximal the Intersection over Union (IoU).

i. Area-ratio judgment. Calculate the pixel area of all contiguous regions. If the area of the second largest contiguous region is ≥ 0.5 times the area of the largest region, directly adopt global fitting (fit an ellipse to all activated pixels as a single cluster). This rule prevents fragments that should belong to the same cluster from being split into independent fits.

ii. Shape similarity judgment. If the area proportion condition was not satisfied, the parameter differences between the ellipse fitted to the largest region and the globally fitted ellipse were calculated separately: the Euclidean distance between the two ellipse centers, d_{center}, and difference in major-to-minor axis ratios, $\left|\frac{a_{global}}{b_{global}} - \frac{a_{max}}{b_{max}}\right|$. If d_{center} >12 μm and the axis ratio difference > 0.3, the two ellipses were considered to have excessively large shape discrepancy, demonstrating that smaller contiguous region have a relatively significant impact on the shape of the RF distribution. In this case, the globally fitted ellipse was directly adopted; otherwise, proceed to the next step.

iii. IoU-based optimal selection. Calculate the IoU values of both the ellipse fitted to the largest region and the globally fitted ellipse. Select the one with the larger IoU as the final fitting result.

The quality of ellipse fitting was quantified with the IoU metric, defined as

$$IoU = \frac{Area(E \cap A)}{Area(E \cup A)}$$

where E is the fitted ellipse and A is the actual activated RF region. IoU ranges from zero to one, with higher values indicating better agreement.

Stimulation Selectivity under DVC Conditions

We employed a simple measure to evaluate the selectivity of DVC stimulation. Specifically, we calculated the ratio of mean activation thresholds of RGCs inside the target region versus those in the surrounding region, termed RTH (ratio of thresholds inside versus outside the target region). The target region is defined as a circular area centered at the center of the fitted RF ellipse with a radius R equal to the semi-major axis length of the ellipse. The surrounding region is defined as an annular area with inner radius R and outer radius $\sqrt{2}R$, as illustrated in Figure S6a. This range ratio ensures that the two regions cover the same area, thereby reducing statistical error. Our results show that the mean activation threshold of RGCs within the target region is **0.86–0.94 times** that of RGCs in the surrounding ring region. Moreover, the RTH values are generally lower for the higher target RF activation area condition (DVC3000). These results indicate DVC achieves good selectivity for activating the target region.

Table S1. RF centroid position, activated area at 1.1×STH, and linear fitting parameters (K and R^2) of area versus intensity under static virtual channel (SVC) stimulation with varying current ratio α.

α	Minimum Centroid Y (μm)	Maximum Centroid Y (μm)	Range of Centroid Y (μm)	RF Area at 1.1×STH (μm^2)	K	R^2
0	-38.54	-30.41	8.13	2014.29	18,159.19	0.976
0.1	-38.54	-30.20	8.34	2187.71	20,300.83	0.984
0.2	-34.22	-23.41	10.80	2491.22	25,044.90	0.992
0.3	-23.30	-15.65	7.65	2739.06	32,445.20	0.994
0.4	-15.97	-6.82	9.15	3991.26	35,361.16	0.982
0.5	-2.83	2.60	5.43	5576.78	35,503.89	0.986
0.6	3.44	17.91	14.46	4302.27	35,316.96	0.987
0.7	15.86	26.96	11.10	3376.34	32,282.38	0.983
0.8	22.31	31.06	8.74	2940.99	28,424.68	0.975
0.9	27.89	33.05	5.16	2870.34	22,497.19	0.998
1	31.49	37.02	5.53	2569.89	20,124.54	0.985

For each α condition, the stimulus intensity was varied from 1.05×STH to 1.35×STH (in 0.05×STH increments). The linear fits were performed over this range, with the slope K representing the average area increase per 1×STH intensity rise ($R^2 > 0.975$ for all α).

Table S2. Activated RF area of RGCs under DVC stimulation at different inter-virtual–channel intervals (ΔT) and stimulus intensities, expressed as absolute values and as percentages of the optimal SVC union area.

	ΔT (ms)	0.85×IB_i (μm²/ %)		0.9×IB_i (μm²/ %)		0.95×IB_i (μm²/ %)		1×IB_i (μm²/ %)	
	0.1	3630	**82**	5286	120	6887	156	9560	217
	1	3878	**88**	6304	143	7642	174	10,125	230
	10	1632	**37**	3878	**88**	5640	128	7929	180
$DVCP_1$	20	899	**20**	2962	**67**	4471	102	7730	176
	30	560	**13**	1632	**37**	4449	101	5813	132
	50	246	**6**	899	**20**	3295	**75**	4845	110
	100			899	**20**	2903	**66**	4415	100
	0.1	4204	**88**	6591	138	9156	192	11,637	244
	1	5793	121	6713	141	9171	192	11,819	248
	10	1575	**33**	5864	123	7324	154	9171	192
$DVCP_2$	20			3024	**63**	5866	123	7418	156
	30			246	**5**	5247	110	6611	139
	50					3024	**63**	5983	125
	100					3024	**63**	5343	112
	0.1	3759	**96**	4710	120	8417	215	10,030	256
	1	3779	**96**	5682	145	8450	215	10,241	261
	10	2244	**57**	3934	100	5682	145	6568	167
$DVCP_3$	20	1068	**27**	2831	**72**	4475	114	6472	165
	30			2230	**57**	3777	**96**	4944	126
	50			1590	**41**	3116	**79**	4790	122
	100			1068	**27**	3112	**79**	4443	113

Empty cells indicate that no neurons were activated. Bold values indicate DVC activated area smaller than the optimal SVC union area.

Table S3. The literature directly relevant to retinal prostheses (2023 onwards).

Prosthesis / Study	Functional Outcomes	Major Limitations Mentioned
**Argus II** (Shirian et al., 2025, Titchener et al., 2023)	Able to recognize single flickering objects. Able to detect motion direction. Improved orientation, mobility, and object recognition.	**Adverse events:** Adverse events, device performance issues, and cognitive challenges emerged during long-term use. **Resolution limitations:** Low resolution and narrow field of view differ markedly from natural vision. **Inter-individual variability:** Substantial variability in outcomes exists among patients.
**PRIMA** (Holz et al., 2026, Muqit et al., 2024)	Able to concurrently utilize prosthetic central vision and natural peripheral vision. Restores vision in atrophic retinal regions without compromising residual natural vision.	**Implant misplacement:** Implant misplacement is a critical challenge affecting visual outcomes. **Surgical complications:** One surgical complication was reported.
**44-Channel Suprachoroidal Prosthesis** (Petoe et al., 2025)	Enhanced spatial localization, motion discrimination, orientation, mobility, and quality of life. Consistent improvement in functional visual tasks and mobility in everyday environments.	**Inter-individual variability:** Individual outcomes exhibit variability. **Inconsistent spatial mapping:** One subject failed to demonstrate coherent spatial mapping.

Table S4. The literature directly relevant to current steering (2021 onwards).

Keywords	Reference	Relevance and Main Findings
virtual channel, current steering, retinal stimulation, multielectrode stimulation	Vasireddy PK et al. *Cell Rep*. **2026**.	This study employed **biophysical simulations** and revealed the mechanisms underlying linear and nonlinear summation of **multi-electrode stimuli**. The hierarchical approach is methodologically consistent with DVC's two-stage optimization strategy.
	Meikle SJ et al. *J Neural Eng*. **2023**.	This study tested whether **current steering** could manipulate the locus of neural activity across different cortical layers in rat visual cortex, similarly to our examination of how α values influence RGC population activation centroids.
	Meikle SJ et al. *J Neural Eng*. **2022**.	This study validated the controllability of **virtual electrodes** within a plane on the rat visual cortex, highly consistent with the spatial position of RGC RF centroids by varying the current ratio (α) between two electrodes under retinal virtual channel stimulation.
	Meikle SJ et al. *IEEE EMBC*. **2021**.	This conference paper similarly tested **current steering** within a single cortical layer. The analytical approach of quantifying virtual electrode shifts via the centroid position is methodologically consistent with our calculation of activated RGC receptive field centroid displacement.
	Chen ZC et al. *IEEE EMBC*. **2021**.	This study proposed a real-time **current steering** optimization framework based on the principle of electric field superposition. This methodology employs the same linear superposition conditions used in our calculation of electric field distributions under VC stimulation.

Table S5. Summary of publications utilizing single-cell threshold definition.

Literature	Research type	Threshold definition	Stimulus
Jeng et al., 2011, J Neural Eng	Computational modeling	"The threshold for eliciting a propagating action potential was calculated to within 0.1 μA."	200 μs rectangular stimulus pulse via stimulating electrode fixed at 25 μm from the axon
Tsai et al., 2012, PLOS ONE	Computational modeling	"The threshold is defined as the lowest stimulus current that elicited an RGC action potential. "	Cathodic-first biphasic stimulus via a disk electrode from the vitreous-side 40 mm axial distance above the RGC somatic center
Kish et al., 2023, J Neural Eng	Computational modeling	"We calculated the action potential threshold at each electrode location using a bisection algorithm (with convergence of 0.1 μA)."	Biphasic, cathodic-first pulse, 0.45 ms per phase
Song et al., 2024, Int J Neural Syst	Computational modeling	"The definition of population threshold was the minimum RGC threshold found in the activated group."	Temporal interference and sinusoidal stimulus via extra-ocular electrode array
Jensen et al., 2006, Exp Eye Res	in vitro (rabbit retina)	"Threshold currents were determined by increasing a subthreshold current until action potentials were elicited more than 50% of the time for five or more consecutive stimulations."	2 Hz monophasic current pulses via a 500 μm diameter platinum wire
Vilkhu et al., 2021, J Neural Eng	In vitro (macaque retina)	"p(a) is the spike probability of a given cell…Fitted sigmoidal curves were used to compute the activation threshold, defined as the current amplitude producing 50% spiking probability."	Charge-balanced triphasic pulse; 50 μs per phase via epiretinal 512-electrode array
Madugula et al., 2023, J Neurosci	In vitro (macaque retina)	"The axon bundle threshold for each stimulating electrode is defined as the lowest current amplitude at which the activity in any off-array cell is observed."	Charge-balanced triphasic pulse; 50 μs per phase via epiretinal 512-electrode array

Table S6. Optimal parameters under SVC and DVC.

Target RF area	Stimulation mode	VC / VC in DVCP	ΔT	Current level	Relative level to best SVC	Relative level to SVC threshold
$1600\mu m^2$	SVC	α_1	-	-	-	-
	DVC	α_1 (in $DVCP_1$)	20ms	11.34μA	0.88	$1.03\times Th_{\alpha 1}$
	SVC	α_2	-	-	-	-
	DVC	α_2 (in $DVCP_1$)	20ms	12.88μA	0.88	**$0.92\times Th_{\alpha 2}$**
		α_2 (in $DVCP_2$)	30ms	13.32μA	0.91	**$0.96\times Th_{\alpha 2}$**
	SVC	α_3	-	-	-	-
	DVC	α_3 (in $DVCP_2$)	30ms	13.32μA	0.91	**$0.97\times Th_{\alpha 3}$**
		α_3 (in $DVCP_3$) *	30ms	12.88μA *	0.88	**$0.94\times Th_{\alpha 3}$**
	SVC	α_4	-	-	-	-
	DVC	α_4 (in $DVCP_3$) *	30ms	11.31μA *	0.88	**$0.99\times Th_{\alpha 4}$**
$3000\mu m^2$	SVC	α_1	-	12.88μA	1.00	$1.17\times Th_{\alpha 1}$
	DVC	α_1 (in $DVCP_1$)	20ms	11.72μA	0.91	$1.06\times Th_{\alpha 1}$
	SVC	α_2	-	14.63μA	1.00	$1.05\times Th_{\alpha 2}$
	DVC	α_2 (in $DVCP_1$)	20ms	13.32μA	0.91	**$0.96\times Th_{\alpha 2}$**
		α_2 (in $DVCP_2$)	100ms	14.20μA	0.97	$1.02\times Th_{\alpha 2}$
	SVC	α_3	-	14.63μA	1.00	$1.07\times Th_{\alpha 3}$
	DVC	α_3 (in $DVCP_2$)	100ms	14.20μA	0.97	$1.04\times Th_{\alpha 3}$
		α_3 (in $DVCP_3$) *	10ms	12.88μA *	0.88	**$0.94\times Th_{\alpha 3}$**
	SVC	α_4	-	12.86μA	1.00	$1.12\times Th_{\alpha 4}$
	DVC	α_4 (in $DVCP_3$) *	10ms	11.31μA *	0.88	**$0.99\times Th_{\alpha 4}$**

* Under both the 1600 μm^2 and 3000 μm^2 target activation area conditions, the same stimulation current was applied. Bold font: Stimulation conditions below the SVC population activation threshold.

Table S7. The literature directly relevant to DVC stimulation, temporal interactions in retinal prostheses, and computational RGC modeling.

Keywords	Reference	Relevance and Main Findings
retinal stimulation, temporal interactions, sequential stimulation, spatial resolution	Ye Z, Chan LLH. *J Neural Eng*. **2025**.	This study demonstrated that **aperiodic retinal stimulation** significantly improved trial-to-trial response consistency and reduced desensitization compared to periodic stimulation. Directly supporting the rationale of using inter-VC interval (ΔT) to improve activation outcomes with DVC.
	Kasowski JM et al. *J Neural Eng*. **2025**.	This study provided behavioral evidence that **structured temporal scheduling** can preserve spatial clarity under safety constraints. Directly related to the improvement in retinal VC stimulation outcomes achieved by DVC through regulating the ΔT parameter.
	Wang HZ, Wong YT. *IEEE Trans Neural Syst Rehabil Eng*. **2025**.	This study confirmed that **structured temporal sequencing** alone can enhance perceptual grouping and task efficiency compared to simultaneous presentation, relating to the sequential delivery of VC stimuli in DVC.
	Muralidharan M et al. *J Neural Eng.* **2024**.	This study systematically evaluated how RGCs respond to continuous, gap-free frequency-varying pulse trains. The experimental paradigm of systematically investigating how **temporal continuity of stimulation** affects neural responses is methodologically similar to systematic parametric scanning of inter-VC intervals (ΔT) in DVC.
	Shah NP et al. *eLife*. **2024**.	This study proposed a strategy of decomposing complex continuous stimuli into simple stimulus sequences and achieving **precise control through temporal optimization** is conceptually parallel to DVC's approach of decomposing continuous virtual channel activation into discrete VC pair sequences and modulating RGC population responses via ΔT optimization.
	Corna A et al. *J Neural Eng.* **2024**.	This study confirmed that specific **temporal patterns of electrical stimulation** can preferentially activate somata while sparing passing axons, thereby markedly improving stimulation focality. This provides a methodological precedent for DVC research to introduce inter-VC interval (ΔT) as a novel control dimension.

	Kish KE et al. *Sci Rep*. **2023**.	This study revealed that retinal thickness significantly correlates with perceptual threshold, and that inter-individual anatomical differences contribute to variability in electrode performance. This underscores the clinical necessity of the DVC optimization framework, flexible stimulation strategies utilizing temporal tuning are required to achieve individualized, high-precision activation.
	Ahn J et al. *Korean J Physiol Pharmacol*. **2023**.	This study demonstrated that multiple **low-amplitude consecutive pulses increased RGC firing** comparably to single high-amplitude pulses, but with significantly better spatial confinement, directly relating to DVC in the mechanism of using sequential sub-threshold stimuli to achieve focal activation via temporal summation.
computational modeling, RGC, electrical stimulation	Alqahtani AM. *J King Saud Univ Sci*. **2026**.	This study used a **morphologically realistic RGC computational model** to systematically quantify how pulse duration, waveform, and electrode-RGC distance interact to avoid unintended axonal activation, providing a parametric optimization framework analogous to DVC's multi-parameter approach for achieving focal activation.
	Vilkhu RS et al. *J Neural Eng*. **2025**.	This study used a **biophysical model** to investigate how multi-electrode currents sum linearly or nonlinearly depending on electrode placement relative to RGC axon and soma, directly relating to current ratio between electrode pair (α) which governs the electric field distribution.
	Fine I, Boynton GM. *Sci Rep*. **2024**.	This study established a biophysically realistic V1 model and revealed that **spatial resolution** of cortical prostheses is constrained by neurophysiological rather than engineering constraints, supporting the need for stimulation strategies like DVC that optimize activation within neurophysiological limits.
	Kish KE et al. *J Neural Eng*. **2023**.	This study provided a comprehensive methodological guide for constructing **multi-compartment RGC cable models** and applying extracellular stimuli, including sensitivity analyses of morphological parameters that directly related to the RGC modeling framework used in our simulations.
	Ghaffari DH et al. *IEEE EMBC*. **2021**.	This study developed a closed-loop optimization framework using data-driven models to find stimulation parameters that produce **focal RGC activation**, offering a methodological parallel to DVC's hierarchical parameter optimization approach for achieving spatially precise responses.
virtual channel, current steering, retinal stimulation, multielectrode stimulation	Vasireddy PK et al. *Cell Rep*. **2026**.	This study employed **biophysical simulations** and revealed the mechanisms underlying linear and nonlinear summation of **multi-electrode stimuli**. The hierarchical approach is methodologically consistent with DVC's two-stage optimization strategy.

Meikle SJ et al. *J Neural Eng*. **2023**.	This study tested whether **current steering** could manipulate the locus of neural activity across different cortical layers in rat visual cortex, similarly to our examination of how α values influence RGC population activation centroids.
Meikle SJ et al. *J Neural Eng*. **2022**.	This study validated the controllability of **virtual electrodes** within a plane on the rat visual cortex, highly consistent with the spatial position of RGC RF centroids by varying the current ratio (α) between two electrodes under retinal virtual channel stimulation.
Meikle SJ et al. *IEEE EMBC*. **2021**.	This conference paper similarly tested **current steering** within a single cortical layer. The analytical approach of quantifying virtual electrode shifts via centroid position is methodologically consistent with our calculation of activated RGC receptive field centroid displacement.
Chen ZC et al. *IEEE EMBC*. **2021**.	This study proposed a real-time **current steering** optimization framework based on the principle of electric field superposition. This methodology employs the same linear superposition conditions used in our calculation of electric field distributions under VC stimulation.

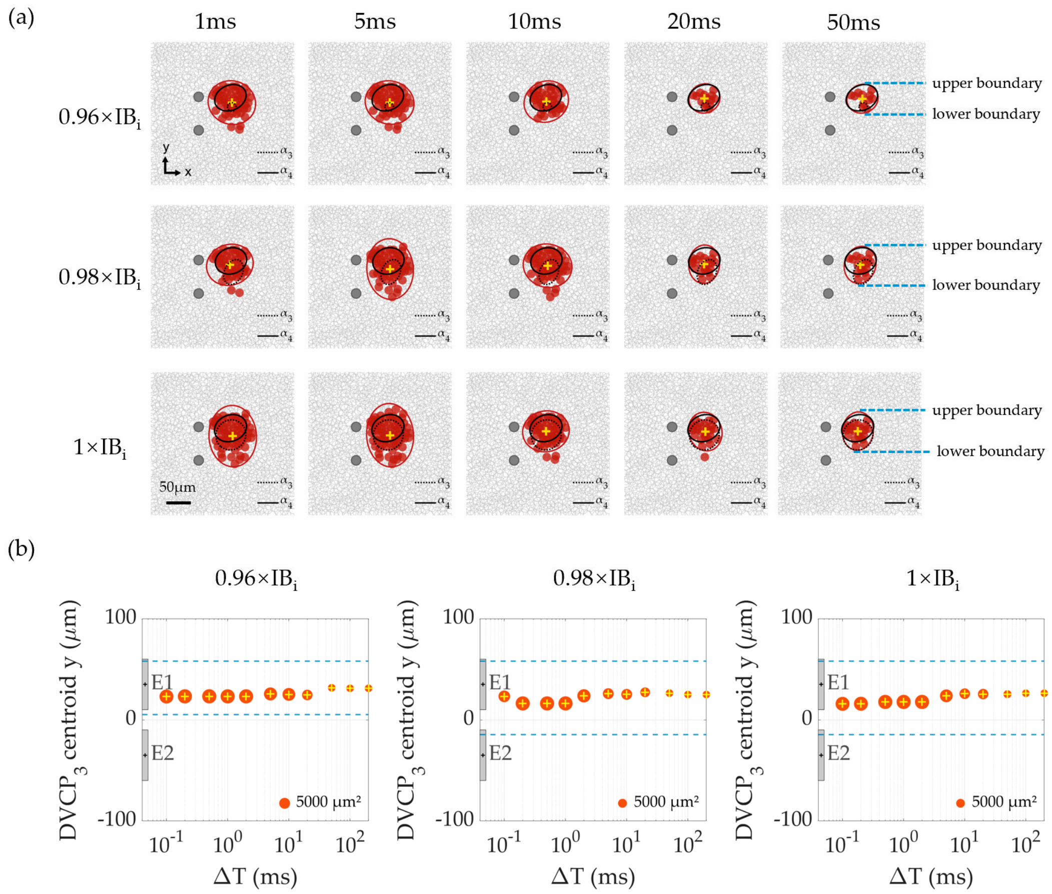

Figure S1. DVCP$_3$-activated RGC RFs across different current intensity and ΔT. (a) Activated RFs under DVCP$_3$ stimulation at ΔT = 1, 5, 10, 20 and 50ms. The activated RF areas were calculated from fitted ellipse areas. Elliptical fits for DVCP$_3$-activated RFs are shown in red, and yellow crosses indicate RF centroids. Black dashed and solid ellipses show the fitted RFs elicited by SVC stimulations at the same current intensity for each constituent α. Red solid circles represent the RFs of the activated RGCs, while inactivated RGCs are indicated in gray open circles. (b) ΔT-dependent changes in activated RF centroid and area under DVC stimulation. Yellow crosses indicate RF centroid positions, the size of the red circles indicates RF area, and the blue dashed lines correspond to the upper and lower boundaries of the SVC superimposed RF activation area identified in panel (a).

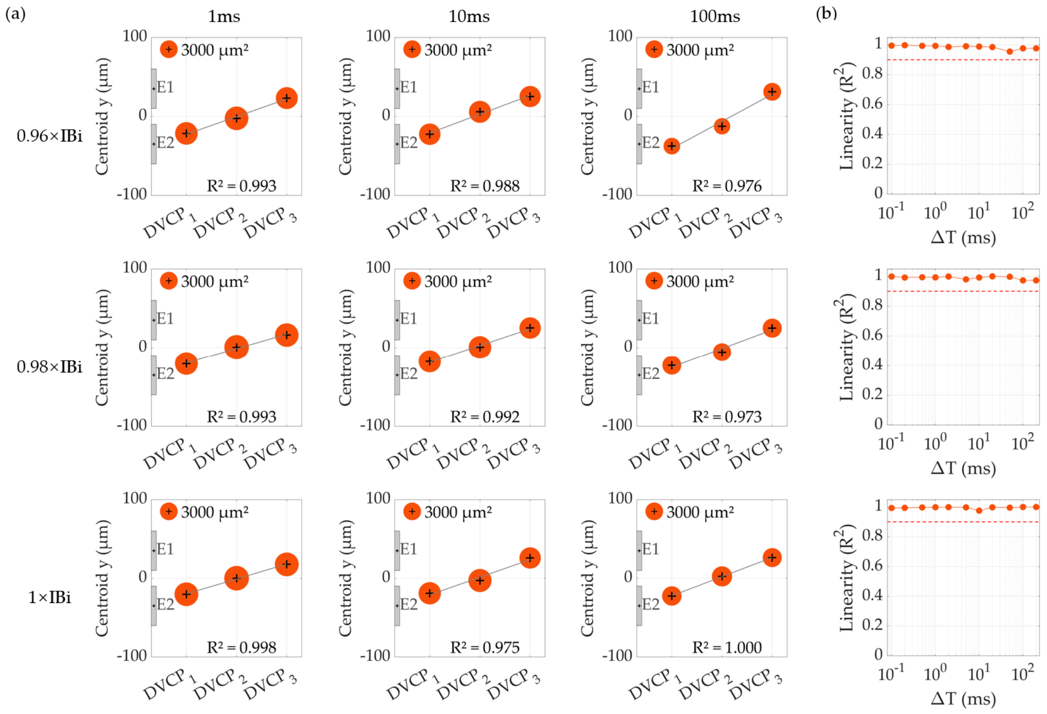

Figure S2. Evaluation of RF centroid displacement linearity under DVC stimulation. (a) Linearity of RF centroid movement with DVCP under DVC stimulation. Black crosses indicate RF centroid positions, the size of red circles indicates RF area, and the gray line is the linear fit of the centroid as a function of DVCP. (b) ΔT-dependent linearity of RF centroid movement with DVCP. The red dashed line indicates the position of $R^2 = 0.9$.

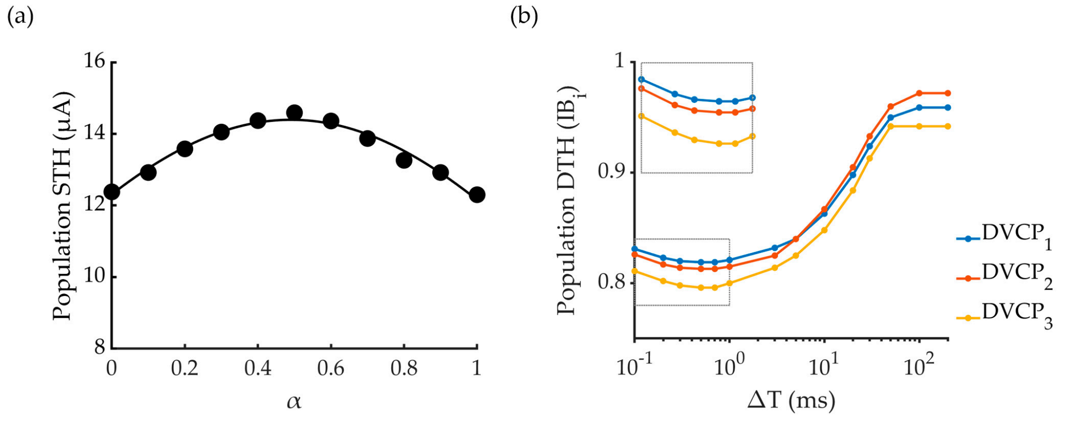

Figure S3. SVC and DVC population thresholds under multi-cell criterion. (**a**) Population activation thresholds in SVC stimulation (STH) as a function of α. Thresholds were defined as the minimal total current injected into the electrode pair required to activate the first ten RGCs. The black curve shows Gaussian fitting of the data points. (**b**) RGC population activation thresholds under DVC stimulation. Thresholds were defined as the minimum IB_i multiple required to activate the first 10 RGCs at the corresponding ΔT when delivered through the electrode pair. Enlarged view of populational activation thresholds under DVC from 0.1 to 1ms (indicated by gray box).

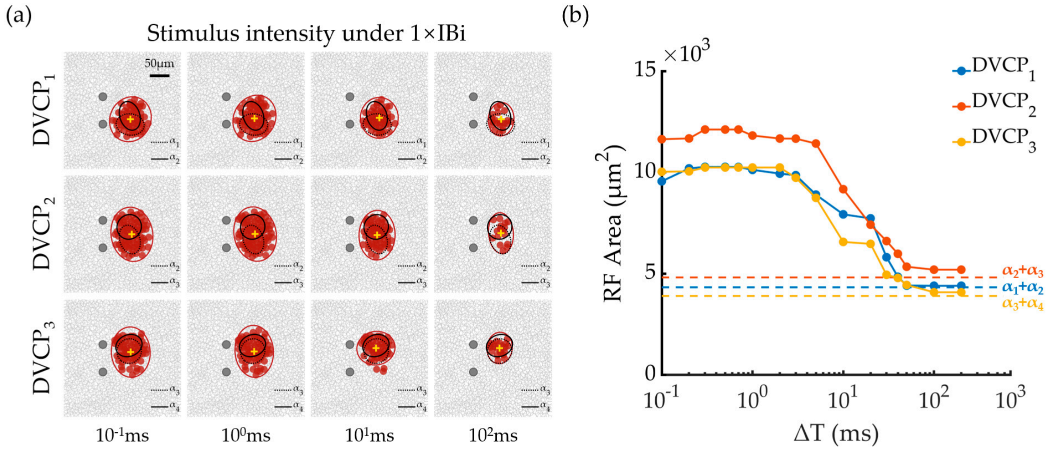

Figure S4. Activated RGC RFs under DVC stimulation with current intensity at 1×IB$_i$. (**a**) Activated RFs under DVC stimulation at ΔT = 0.1, 1, 10, and 100ms. The activated RF areas were calculated from fitted ellipse areas. Elliptical fits for DVCP activated RFs are shown in red, and yellow crosses indicate RF centroids. Black dashed and solid ellipses show the fitted RFs elicited by SVC stimulations at the same current intensity (1×IB$_i$) for each constituent α. Red solid circles represented the RFs of the activated RGCs, while inactivated RGCs were indicated in gray open circles. (**b**) ΔT-dependent changes in activated RF areas under DVC stimulation. Colored dashed lines indicate the union of RF areas from corresponding SVC stimulations at 1×IB$_i$ (i=1,2,3 and 4 for α_1, α_2, α_3 and α_4, respectively).

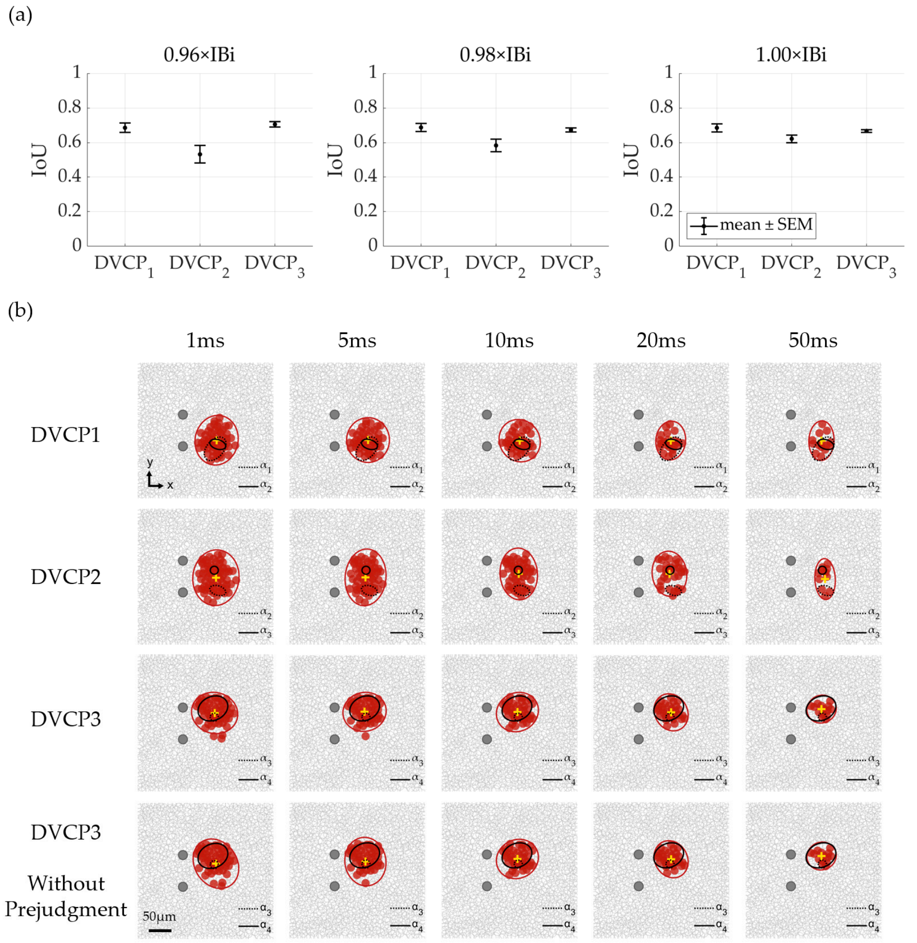

Figure S5. RF ellipse fitting performance. (a) Fitting performance of RF ellipses under different stimulus intensities and DVCP conditions. The data points show the mean and standard error of the IoU data under all ΔT conditions (0.1, 0.2, 0.3, 0.5, 0.7, 1, 2, 3, 5, 10, 20, 30, 40, 50, 100 and 200 ms). (b) Fitting results at 0.96×IB_i. Elliptical fits for DVCP activated RFs are shown in red, and yellow crosses indicate RF centroids. Black dashed and solid ellipses show the fitted RFs elicited by SVC stimulations at the same current intensity (0.96×IB_i) for each constituent α. Red solid circles represented the RFs of the activated RGCs, while inactivated RGCs were indicated in gray open circles.

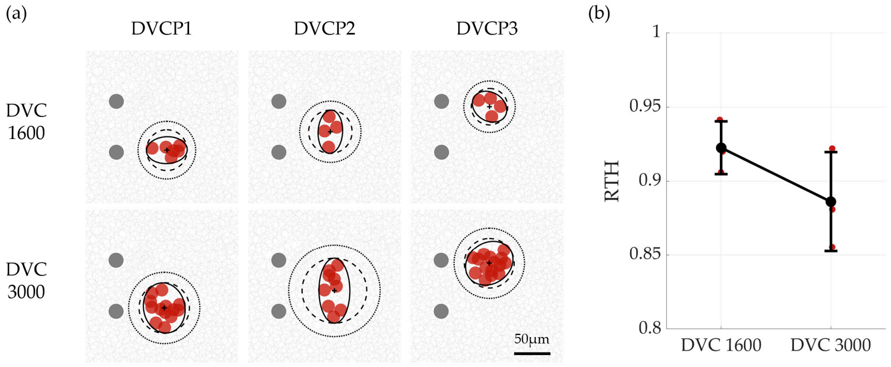

Figure S6. Activation selectivity measurement under DVC conditions. (a) Schematic diagram of ellipse fitting results of activated RFs (black solid line), target region boundary (sparse black dashed line) and outer region boundary (dense black dashed line) on optimal DVC conditions, with target activation areas of 1600 μm² (DVC1600) and 3000 μm² (DVC3000). (b) Ratio of mean activation thresholds of RGCs within the target region to those in the outer region.

References

1. Kasowski, J.M.; Varshney, A.; Sadeghi, R.; Beyeler, M. Simulated prosthetic vision confirms checkerboard as an effective raster pattern for epiretinal implants. *J. Neural Eng.* **2025**, *22*, 046017. https://doi.org/10.1088/1741-2552/adecc4.
2. Wang, H.Z.; Wong, Y.T. Raster Scanning Can Improve Task Performance in Simulated Prosthetic Vision. *IEEE Trans. Neural Syst. Rehabil. Eng.* **2025**, *33*, 4159–4170. https://doi.org/10.1109/tnsre.2025.3617891.
3. Vilkhu, R.S.; Madugula, S.S.; Grosberg, L.E.; Gogliettino, A.R.; Hottowy, P.; Dabrowski, W.; Sher, A.; Litke, A.M.; Mitra, S.; Chichilnisky, E.J. Spatially patterned bi-electrode epiretinal stimulation for axon avoidance at cellular resolution. *J. Neural Eng.* **2021**, *18*, 066007. https://doi.org/10.1088/1741-2552/ac3450.
4. Corna, A.; Cojocaru, A.-E.; Bui, M.T.; Werginz, P.; Zeck, G. Avoidance of axonal stimulation with sinusoidal epiretinal stimulation. *J. Neural Eng.* **2024**, *21*, 026036. https://doi.org/10.1088/1741-2552/ad38de.
5. Muralidharan, M.; Guo, T.; Tsai, D.; Lee, J.-I.; Fried, S.; Dokos, S.; Morley, J.W.; Lovell, N.H.; Shivdasani, M.N. Neural activity of retinal ganglion cells under continuous, dynamically-modulated high frequency electrical stimulation. *J. Neural Eng.* **2024**, *21*, 015001. https://doi.org/10.1088/1741-2552/ad2404.
6. Ahn, J.; Yoo, Y.; Goo, Y.S. Multiple consecutive-biphasic pulse stimulation improves spatially localized firing of retinal ganglion cells in the degenerate retina. *Korean J. Physiol. Pharmacol.* **2023**, *27*, 541–553. https://doi.org/10.4196/kjpp.2023.27.6.541.
7. Shirian, J.D.; Yuan, A.; Rachitskaya, A. Long-Term Outcomes and Patient Experiences with the Argus II Retinal Prosthesis System. *J. Vitreoretin. Dis.* **2025**, *10*, 98–102. https://doi.org/10.1177/24741264251376042.
8. Titchener, S.A.; Goossens, J.; Kvansakul, J.; Nayagam, D.A.X.; Kolic, M.; Baglin, E.K.; Ayton, L.N.; Abbott, C.J.; Luu, C.D.; Barnes, N.; et al. Estimating Phosphene Locations Using Eye Movements of Suprachoroidal Retinal Prosthesis Users. *Transl. Vis. Sci. Technol.* **2023**, *12*, 20. https://doi.org/10.1167/tvst.12.3.20.
9. Holz, F.G.; Le Mer, Y.; Muqit, M.M.K.; Hattenbach, L.-O.; Cusumano, A.; Grisanti, S.; Kodjikian, L.; Pileri, M.A.; Matonti, F.; Souied, E.; et al. Subretinal Photovoltaic Implant to Restore Vision in Geographic Atrophy Due to AMD. *N. Engl. J. Med.* **2026**, *394*, 232–242. https://doi.org/10.1056/NEJMoa2501396.
10. Muqit, M.M.K.; Mer, Y.L.; Koo, L.O.d.; Holz, F.G.; Sahel, J.A.; Palanker, D. Prosthetic Visual Acuity with the PRIMA Subretinal Microchip in Patients with Atrophic Age-Related Macular Degeneration at 4 Years Follow-up. *Ophthalmol. Sci.* **2024**, *4*, 100510. https://doi.org/10.1016/j.xops.2024.100510.
11. Petoe, M.A.; Abbott, C.J.; Titchener, S.A.; Kolic, M.; Kentler, W.G.; Nayagam, D.A.X.; Baglin, E.K.; Kvansakul, J.; Barnes, N.; Walker, J.G.; et al. A Second-Generation (44-Channel) Suprachoroidal Retinal Prosthesis: A Single-Arm Clinical Trial of Feasibility. *Ophthalmol. Sci.* **2025**, *5*, 100525. https://doi.org/10.1016/j.xops.2024.100525.
12. Vasireddy, P.K.; Vilkhu, R.S.; Lotlikar, A.; Brown, J.B.; Phillips, A.J.; Gogliettino, A.R.; Hays, M.R.; Baum, C.; Kato, E.J.; Sharon, A.; et al. Leveraging current steering and the biophysics of spike generation for cellular-resolution electrical stimulation of neurons. *Cell Rep.* **2026**, *45*, 116917. https://doi.org/10.1016/j.celrep.2025.116917.
13. Meikle, S.J.; Hagan, M.A.; Price, N.S.C.; Wong, Y.T. Cortical layering disrupts multi-electrode current steering. *J. Neural Eng.* **2023**, *20*, 036031. https://doi.org/10.1088/1741-2552/acdaf7.
14. Meikle, S.J.; Hagan, M.A.; Price, N.S.C.; Wong, Y.T. Intracortical current steering shifts the location of evoked neural activity. *J. Neural Eng.* **2022**, *19*, 035003. https://doi.org/10.1088/1741-2552/ac77bf.
15. Meikle, S.J.; Ann Hagan, M.; Chiang Price, N.S.; Tat Wong, Y. Filling in the Visual Gaps: Shifting Cortical Activity using Current Steering. In Proceedings of the 2021 43rd Annual International

Conference of the IEEE Engineering in Medicine & Biology Society (EMBC), Mexico, 1–5 November 2021; pp. 5733–5736.
16. Chen, Z.C.; Wang, B.-Y.; Palanker, D. Real-Time Optimization of the Current Steering for Visual Prosthesis. In Proceedings of the 2021 10th International IEEE/EMBS Conference on Neural Engineering (NER), Italy, 4–6 May 2021; pp. 592–596.
17. Jeng, J.; Tang, S.; Molnar, A.; Desai, N.J.; Fried, S.I. The sodium channel band shapes the response to electric stimulation in retinal ganglion cells. *J. Neural Eng.* **2011**, *8*, 036022. https://doi.org/10.1088/1741-2560/8/3/036022.
18. Tsai, D.; Chen, S.; Protti, D.A.; Morley, J.W.; Suaning, G.J.; Lovell, N.H. Responses of Retinal Ganglion Cells to Extracellular Electrical Stimulation, from Single Cell to Population: Model-Based Analysis. *PLoS ONE* **2012**, *7*, e53357. https://doi.org/10.1371/journal.pone.0053357.
19. Kish, K.E.; Lempka, S.F.; Weiland, J.D. Modeling extracellular stimulation of retinal ganglion cells: Theoretical and practical aspects. *J. Neural Eng.* **2023**, *20*, 026011. https://doi.org/10.1088/1741-2552/acbf79.
20. Song, X.; Guo, T.; Ma, S.; Zhou, F.; Tian, J.; Liu, Z.; Liu, J.; Li, H.; Chen, Y.; Chai, X.; et al. Spatially Selective Retinal Ganglion Cell Activation Using Low Invasive Extraocular Temporal Interference Stimulation. *Int. J. Neural Syst.* **2024**, *35*, 2450066. https://doi.org/10.1142/s0129065724500667.
21. Jensen, R.J.; Rizzo, J.F. Thresholds for activation of rabbit retinal ganglion cells with a subretinal electrode. *Exp. Eye Res.* **2006**, *83*, 367–373. https://doi.org/10.1016/j.exer.2006.01.012.
22. Madugula, S.S.; Vilkhu, R.; Shah, N.P.; Grosberg, L.E.; Kling, A.; Gogliettino, A.R.; Nguyen, H.; Hottowy, P.; Sher, A.; Litke, A.M.; et al. Inference of Electrical Stimulation Sensitivity from Recorded Activity of Primate Retinal Ganglion Cells. *J. Neurosci.* **2023**, *43*, 4808–4820. https://doi.org/10.1523/jneurosci.1023-22.2023.
23. Ye, Z.; Chan, L.L.H. Effectiveness of aperiodic retinal stimulation in improving temporal visual cortical response. *J. Neural Eng.* **2025**, *22*, 026062. https://doi.org/10.1088/1741-2552/adc83c.
24. Shah, N.P.; Phillips, A.; Madugula, S.; Lotlikar, A.; Gogliettino, A.R.; Hays, M.R.; Grosberg, L.; Brown, J.; Dusi, A.; Tandon, P.; et al. Precise control of neural activity using dynamically optimized electrical stimulation. *eLife* **2024**, 13, e83424. https://doi.org/10.7554/eLife.83424.
25. Kish, K.E.; Yuan, A.; Weiland, J.D. Patient-specific computational models of retinal prostheses. *Sci. Rep.* **2023**, 13, 22271. https://doi.org/10.1038/s41598-023-49580-6.
26. Alqahtani, A.M. Optimizing retinal prosthesis stimulation: Enhancing the avoidance of unintended axonal activation. *J. King Saud Univ.—Sci.* **2026**, 38, 13612025.
27. Vilkhu, R.S.; Vasireddy, P.K.; Kish, K.E.; Gogliettino, A.R.; Lotlikar, A.; Hottowy, P.; Dabrowski, W.; Sher, A.; Litke, A.M.; Mitra, S.; et al. Understanding responses to multi-electrode epiretinal stimulation using a biophysical model. *J. Neural Eng.* **2025**, 22, 016010. https://doi.org/10.1088/1741-2552/ada1fe.
28. Fine, I.; Boynton, G.M. A virtual patient simulation modeling the neural and perceptual effects of human visual cortical stimulation, from pulse trains to percepts. *Sci. Rep.* **2024**, 14, 17400. https://doi.org/10.1038/s41598-024-65337-1.
29. Ghaffari, D.H.; Chang, Y.-C.; Mirzakhalili, E.; Weiland, J.D. Closed-loop Optimization of Retinal Ganglion Cell Responses to Epiretinal Stimulation: A Computational Study. In Proceedings of the 2021 10th International IEEE/EMBS Conference on Neural Engineering (NER), Italy, 4–6 May 2021; pp. 597–600.
